# The association between bone density evaluated on CT or MRI versus the gold standard dual-energy X-ray absorptiometry (DEXA) in the lumbar spine and hip: a meta-analysis

**DOI:** 10.1016/j.bas.2026.106246

**Published:** 2026-07-25

**Authors:** Luc W.F. van Haaster, Erem Tutal, Rania A. Mekary, Carmen L.A. Vleggeert-Lankamp

**Affiliations:** a Department of Neurosurgery, Leiden University Medical Center, Albinusdreef 2, Leiden, ZA, 2333, the Netherlands; b Computational Neuroscience Outcomes Center at Harvard, Department of Neurosurgery, Brigham and Women's Hospital, 75 Francis St, Boston, MA, 02115, United States of America; c Department of Pharmaceutical Business and Administrative Sciences, School of Pharmacy, MCPHS University, 179, Longwood Ave, Boston, MA, 02115, United States of America; d Department of Neurosurgery, Spaarne Gasthuis Hospital, Spaarnepoort 1, Hoofddorp, TM, 2134, the Netherlands

**Keywords:** Bone mineral density, Hounsfield unit, Vertebral bone quality score, Osteoporosis

## Abstract

**Introduction:**

CT-based Hounsfield units (HU) and MRI-based vertebral bone quality (VBQ)-scores are opportunistic markers of bone mineral density and potential alternatives for dual-energy X-ray absorptiometry (DEXA).

**Research question:**

What is the correlation between HU values and VBQ-scores in relation to DEXA, and what is the corresponding area-under-the-curve (AUC)-based diagnostic discrimination to detect osteopenia and osteoporosis?

**Material and methods:**

PubMed, Embase, Web of Science, Cochrane Library, and CINAHL were searched through August 2025 for studies reporting correlations between DEXA and CT and/or MRI or the AUC-based diagnostic discrimination of CT and/or MRI to detect osteopenia and osteoporosis. Random effects models were used to calculate pooled correlation coefficients and AUC values along with 95% confidence intervals.

**Results:**

Forty-three studies, including 8222 patients, were analyzed. HU demonstrated stronger correlations with DEXA T-scores than the VBQ-score (maximum correlation coefficient: 0.741 (95% CI: 0.578-0.847) vs. −0.476 (95% CI: −0.548 to −0.398) and greater AUC-based diagnostic discrimination for osteopenia and osteoporosis (maximum AUC: 0.875 (95% CI: 0.841-0.909) vs. 0.806 (95% CI: 0.684-0.929) across all vertebrae and DEXA sites. Correlation and AUC-based diagnostic discrimination of HU were consistent across vertebrae, with slightly better results at L2 and the mean of L1–L4. VBQ-score correlations were highest at L1 and gradually decreased from L1 to L4.

**Discussion and conclusion:**

When CT is available, HU appears to be the better-supported opportunistic marker for low BMD than VBQ. All vertebrae demonstrated comparable correlation and AUC-based diagnostic discrimination, with L2 HU and the mean of L1-L4 HU showing slightly superior results.

## Highlights

• HU appears to outperform the VBQ-score across all vertebrae and DEXA sites.
• Correlation and diagnostic performance of HU were consistent across vertebrae.
• VBQ-score correlations were highest at L1 and gradually decreased from L1 to L4.

## 1 Introduction

Osteoporosis is a systemic bone disorder characterized by low bone mass and deteriorated microarchitecture of the trabecular bone (Akkawi and Zmerly, 2018; Föger-Samwald et al., 2020). The prevalence of osteoporosis and the accompanying financial burden are expected to further increase in the future, due to an ageing population (Hernlund et al., 2013; Rachner et al., 2011). Preoperative detection and treatment of osteoporosis are important in spinal surgery patients, as low bone mineral density (BMD) is associated with complications such as pedicle screw loosening and cage subsidence, which can compromise surgical outcomes and necessitate revision surgery (Galbusera et al., 2015; Cho et al., 2018; Khalid et al., 2020). Despite the clinical relevance and rising prevalence, a recent Dutch study suggested that a significant number of patients with osteoporosis remains undiagnosed, estimating a treatment gap of 60% to 72% (Dunnewind et al., 2017). The current gold standard for BMD assessment is dual-energy X-ray absorptiometry (DEXA) measured at the hip or femur (No authors listed. World Health Organization, 2003). Additionally, DEXA scans derived from the vertebra of the lumbar spine are more commonly performed (Akkawi and Zmerly, 2018; Föger-Samwald et al., 2020; Hernlund et al., 2013; Rachner et al., 2011; Pennington et al., 2021). According to the World Health Organization (WHO), a T-score ≥ −1 indicates normal bone density, a T-score < −1 and > −2.5 indicates the presence of osteopenia, and a T-score ≤ −2.5 indicates the presence of osteoporosis (No authors listed. World Health Organization, 2003). However, increasing evidence suggests that *in vivo* assessment of bone density using DEXA is inaccurate. Factors such as osteophytes, vascular calcifications, scoliosis, and excessive fat deposits can lead to overestimation of bone density, while variability in the absorptiometry measurement site further limits the generalizability of the results (Liu et al., 1997; Yu et al., 2012; Rand et al., 1997; Pappou et al., 2006). Furthermore, although DEXA is considered to be the gold standard for the diagnosis of osteoporosis, DEXA remains underutilized among eligible patients (Amarnath et al., 2015; Gillespie and Morin, 2017).

Alternative methods to assess the BMD of the lumbar spine have been proposed, such as computed tomography (CT)-based Hounsfield units (HU) and magnetic resonance imaging (MRI)-based vertebral bone quality (VBQ) score (Ahmad et al., 2023; Deshpande et al., 2023; Zhu et al., 2021a; Pinto et al., 2022; Zaidi et al., 2019). One major advantage of these imaging modalities is that CT- and MRI-scans are commonly obtained during the preoperative assessment and can therefore be used opportunistically, as compared to DEXA. HU represents a standardized scale used in CT imaging to quantify the relative radiodensity of tissues and can therefore be used as a measure of bone density (Schreiber et al., 2011; Hendrickson et al., 2018). The VBQ-score assesses the amount of fat infiltration in the vertebral body through T1-weighted MR images of the lumbar spine (Salzmann et al., 2022; Ehresman et al., 2020). The attenuation (i.e., BMD-indicative) values derived from these opportunistic imaging modalities have been shown to correlate with DEXA, but varying sites of measurement and variability in measurement methods are likely to be responsible for the spread in reported correlation coefficients and diagnostic accuracy (ROC-analysis) (Ahmad et al., 2023; Deshpande et al., 2023; Zhu et al., 2021a; Pinto et al., 2022; Zaidi et al., 2019; Chen et al., 2023). Existing meta-analyses comparing BMD measured with DEXA scans and opportunistic methods do not differentiate between specific DEXA-measurement sites or methods and measurement sites (varying lumbar vertebra) of CT/MRI assessments (Zhu et al., 2021b; Ahern et al., 2021). Given that bone density varies across the body and along the vertebral column, it is reasonable to expect that results may differ for each vertebral body and measurement site. This meta-analysis aimed to provide a better understanding of the correlation and area-under-the-curve (AUC)-based diagnostic discrimination of CT-based Hounsfield units and MRI-based VBQ-scores in assessing bone mineral density relative to the current gold standard, DEXA. In addition, the optimal vertebral level for opportunistic assessment of BMD was evaluated. Ultimately, this could support improved screening and diagnosis of osteoporosis, especially in patients undergoing preoperative imaging, enhancing the overall quality of spinal care.

## 2 Material and methods

### 2.1 Search methods for the identification of studies

The PRISMA 2020 27-item checklist was used as the reporting guideline for this review (Page et al., 2021). A systematic search was conducted in five databases of medical literature: PubMed, Embase, Web of Science, Cochrane Library, and CINAHL. The search spanned from inception to August 2025 (Appendix A).

### 2.2 Measurement methods

#### 2.2.1 DEXA

In clinical practice, DEXA measurements are most commonly performed at the femoral neck or total hip and are increasingly obtained at the lumbar spine. The resulting individual T-scores can be used for BMD assessment, but often the lowest T-score is used among the assessed regions.

#### 2.2.2 CT-based HU measurement

Several methods exist for measuring Hounsfield units. Measurements are typically obtained at L1 to L4, and can be performed in the midaxial, midsagittal, and midcoronal plane, with the midaxial plane being most commonly used. With regard to measurement methods, some approaches measure the entire midvertebral body, whereas others use a standardized circular ROI. As HU represents the quantified relative radiodensity of tissues, a higher HU value corresponds to higher bone density.

#### 2.2.3 MRI-based VBQ measurement

The VBQ-score is measured exclusively in the midsagittal plane and most commonly from L1 to L4. As the VBQ-score represents the amount of fat infiltration in the vertebral body, a higher VBQ-score corresponds to lower bone density.

### 2.3 Criteria for considering studies for this review

This systematic review and meta-analysis included retrospective and prospective studies aiming to compare and/or correlate lumbar bone density assessment using conventional CT and/or MRI with DEXA measurements of the lumbar spine, hip, and/or femur. The following inclusion criteria were used: 1) patients aged 18 years or older, 2) sample size larger than 20 patients, 3) conventional CT/MRI protocols used for measuring respectively lumbar HU values and VBQ-scores, 4) DEXA T-score measurement in the lumbar spine, femur or hip, scored according to the WHO-definition of osteoporosis and 5) reporting either the correlation coefficient between CT/MRI attenuation values and DEXA T-scores and/or the receiver operating characteristic (ROC) analysis, including the AUC and corresponding 95% confidence interval (95% CI) or p-value (World Health Organization, 1994). Exclusion criteria included: 1) time between CT/MRI and DEXA measurements longer than 1 year, 2) CT/MRI measurements at non-lumbar spinal sites, such as cervical or thoracic spine, mandibula, tibia, fibula, radius or ulna, 3) study cohort containing patients with previous lumbar fusion, due to the inability to accurately measure CT/MRI attenuation values after lumbar fusion, 4) study cohort containing patients with systemic pathology possibly affecting bone quality (i.e. skeletal hyperostosis, severe adult spinal deformity, and malignancies), except for osteopenia and osteoporosis, 5) study cohort containing patients receiving medication specifically aiming to enhance bone quality, 6) use of contrast on radiological imaging, 7) biomechanical, animal or cadaver studies, 8) meeting abstracts, 9) case reports and 10) duplicate articles. Two researchers (LWFH and ET) independently screened the titles and abstracts of all retrieved records for eligibility, and disagreements were discussed with a third reviewer (CLAVL). Full text screening of remaining records was performed independently by both researchers, and discrepancies were discussed with the third reviewer.

### 2.4 Assessment of risk-of-bias for the included studies

Two researchers (LWFH and ET) independently assessed the risk-of-bias using the Quality Assessment Tool for Diagnostic Accuracy Studies-2 (QUADAS-2), and discrepancies were discussed with a third reviewer (CLAVL) (Whiting et al., 2011). This tool was used to evaluate the risk-of-bias and applicability of diagnostic accuracy studies based on four domains: patient selection, index test (i.e., CT or MRI), reference standard (i.e., DEXA scan) and flow and timing, resulting in low, high or unclear risk-of-bias for each domain and low, high, or unclear applicability concerns for the first three domains. In the patient selection domain, factors such as consecutive patient enrollment, avoidance of a case-control design, and the risk-of-bias associated with inappropriate exclusions were considered. In the flow and timing domain, various factors were evaluated, including whether all patients received the same reference standard and whether all patients were included in the analysis (Appendix B). While the original QUADAS-2 guidance recommends that studies should be at low risk in all domains to be considered high quality, we applied a slightly less stringent criterion, allowing studies with a maximum of one domain at high or unclear risk-of-bias to be classified as high quality, as adapted from Brunelli et al. (2023). Consequently, records identified as having a ‘’high’’ or ‘’unclear’’ risk-of-bias in at least three of the four domains were considered to be of low quality. Studies were deemed to be of high quality with a ‘’low’’ risk-of-bias assessment in at least three of the four domains. Other classification patterns in studies were regarded as moderate quality. The high/moderate/low quality classification is an adapted overall categorization and should not be interpreted as an original QUADAS-2 summary score.

### 2.5 Data collection and analysis

Two researchers (LWFH and ET) extracted the data from all included studies and entered the data into an Excel spreadsheet. The subsequent data were extracted: publication year, study design, country, number of patients, attenuation value protocol, location of CT/MRI- and DEXA-measurements, time between CT/MRI and DEXA, patient characteristics, attenuation values (HU value or VBQ-score), correlation parameters, ROC-analysis outcomes, and other inclusion and exclusion criteria. ROC-analysis is a statistical method used to assess the AUC-based diagnostic discrimination of a test by evaluating its ability to discriminate between different outcomes, such as normal BMD and low BMD, and is typically expressed through a curve that plots sensitivity against 1-specificity. When an article reported multiple measurements at the same vertebral level obtained in different imaging planes (e.g., axial, sagittal, and coronal), measurements from the axial plane were selected for analysis to avoid double-counting of patients. Furthermore, when multiple measurement methods were reported (e.g., region-of-interest and volume-of-interest approaches), the most conventional method (region-of-interest) was selected to enhance the generalizability of the findings and prevent double-counting patients. All extracted data were transferred to the Comprehensive Meta-Analysis version 4 software for data analysis. The pooled correlation coefficients and AUCs were determined using a random-effects model based on the DerSimonian and Laird method and were presented with the 95% CI (DerSimonian and Laird, 1986). The Cochrane Q statistic was used to evaluate the presence of heterogeneity with a significance level of p < 0.10 (Huedo-Medina et al., 2006). Heterogeneity was quantified using the I-squared (I^2^) index, reported as low (0-25%), moderate (26-50%), substantial (51-75%), or high (76-100%). To address heterogeneity, all analyses were stratified by the location of DEXA and the location of the CT/MRI attenuation value. Additionally, correlation analyses were stratified by correlation type (Pearson for normally distributed data vs. Spearman for not normally distributed data), and AUC-analyses were stratified by outcome type (osteopenia and/or osteoporosis). Egger's regression intercept was used to assess potential asymmetry in the funnel plot due to an alleged small-study effect. In case of any asymmetry in the funnel plot proven visually or statistically, the trim-and-fill method was applied to impute potentially missing studies and calculate the adjusted correlation coefficient, contingent on publication bias being the source of asymmetry. Univariate meta-regression on 1) time interval between CT and DEXA imaging, 2) percentage of osteoporotic patients, and 3) study quality was performed when at least ten studies were available for a single outcome to evaluate whether the correlation coefficient varied across the levels of these trial-level covariates. A two-sided p-value <0.05 was considered statistically significant, unless specified otherwise.

## 3 Results

### 3.1 Search and selection results

**Fig. 1:**
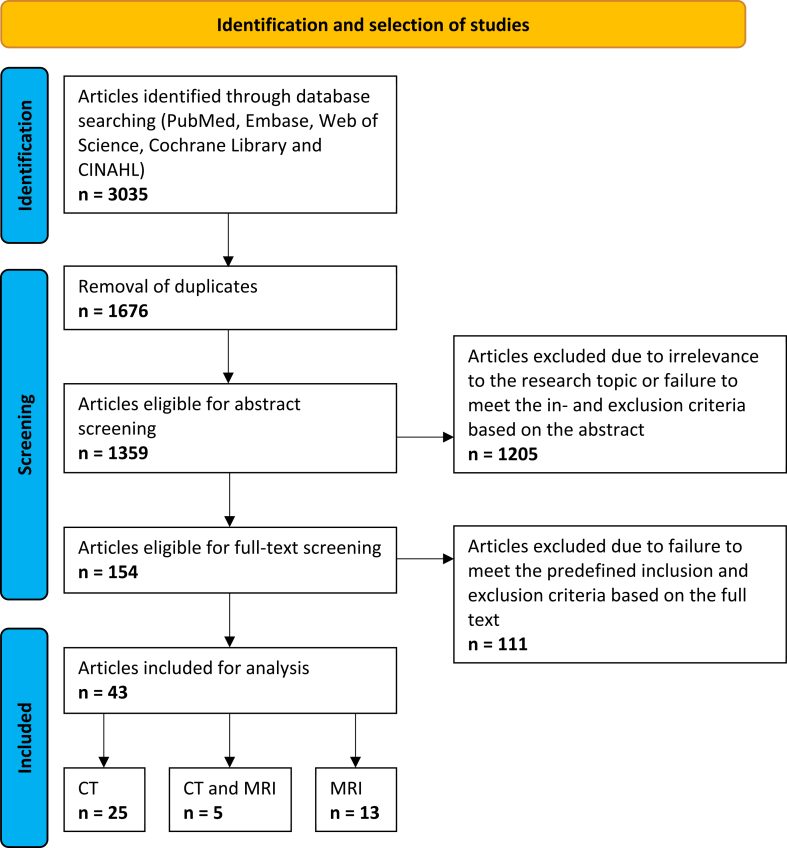
Flow diagram of the identification and selection of studies. Abbreviations: CT; computed tomography, MRI; magnetic resonance imaging.

The initial search yielded a total of 3035 records, of which 1676 duplicates were removed, resulting in 1359 articles eligible for title and abstract screening. A total of 1205 articles were excluded due to irrelevance to the research topic or failure to meet the predefined in- and exclusion criteria based on the abstract, after which full text screening was performed for the remaining 154 articles. Based on the full-text review, 111 articles were excluded due to failure to meet the predefined inclusion and exclusion criteria, resulting in the inclusion of 43 articles in the analysis (Hendrickson et al., 2018; Hiyama et al., 2024, 2025; Zhang et al., 2021, 2023; Wang et al., 2023, 2024; Kim et al., 2019, 2024; Saad et al., 2024; Li et al., 2024a, 2024b, 2025; Yang et al., 2022; Özmen et al., 2023; Viswanathan et al., 2023; Yao et al., 2022; Chang et al., 2021, 2025; Elarjani et al., 2021; Booz et al., 2020; Berger-Groch et al., 2020; Zou et al., 2019; Fujimoto et al., 2024; Zheng et al., 2024; Pu et al., 2023a, 2023b; Courtois et al., 2023; Choi et al., 2016; Xu et al., 2023; Lee et al., 2013, 2015; Vadera et al., 2023; Jameshouran et al., 2020; Nishi et al., 2025; Yurac-Barrientos et al., 2025; Pabbisetti et al., 2025; Su et al., 2025; Yan et al., 2025; Langer et al., 2025; Najafi et al., 2024; Cetik et al., 2025; Khoroushi et al., 2024). An overview of the identification and selection process is provided in Fig. 1.

The 43 analyzed articles included 8222 patients over five prospective cohort studies (n = 417) and 38 retrospective cohort studies (n = 7805). The median of the reported mean ages was 65.8 years (range 48.8-73.2 years). For articles evaluating CT, the mean time between CT and DEXA was 95.2 days, whereas this was 93.4 days for articles examining MRI. An overview of all included articles is provided in Appendix C and Appendix D.

### 3.2 Risk-of-bias assessment

**Fig. 2:**
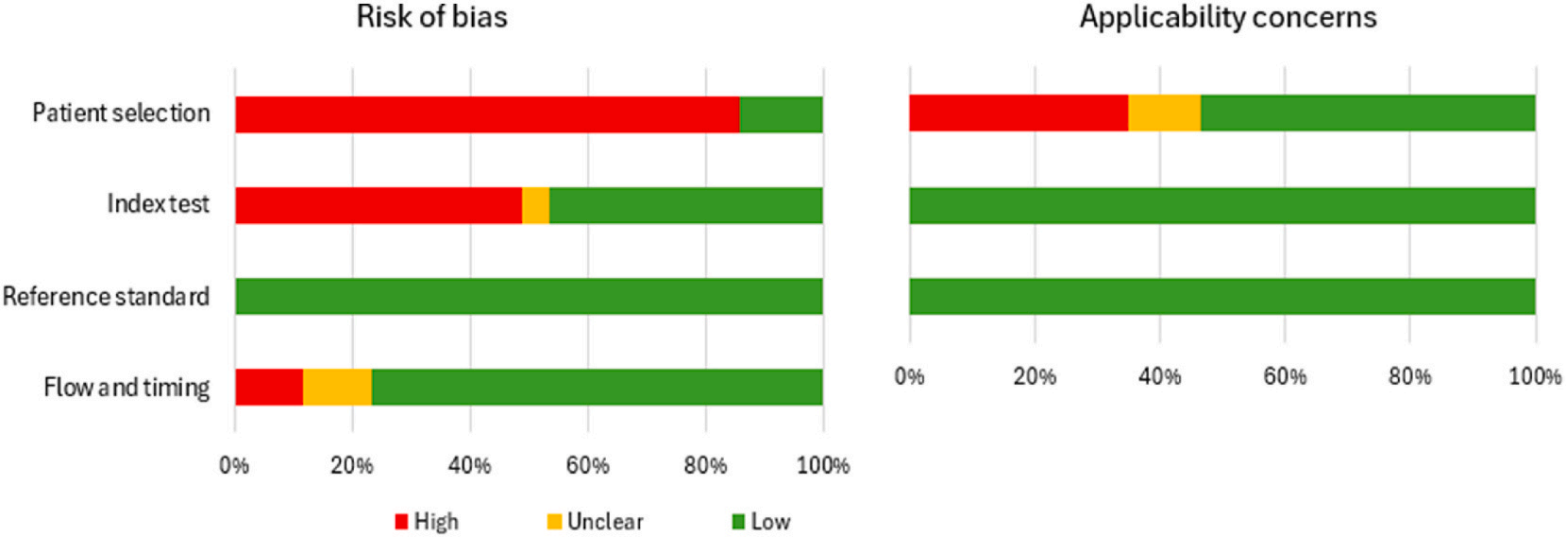
Overview of risk-of-bias and applicability concern assessment, according to the domains of the QUADAS-2 tool, presented as total percentage.

All 43 included articles were evaluated for risk-of-bias using the QUADAS-2 tool. Twenty articles were classified as high quality. Eighteen articles were of moderate quality. Five studies were categorized as low quality. For both the risk of bias assessment as the applicability concerns, the domain that most frequently raised concerns was patient selection. The results are illustrated in Table 1 and Fig. 2.

**Table 1:**
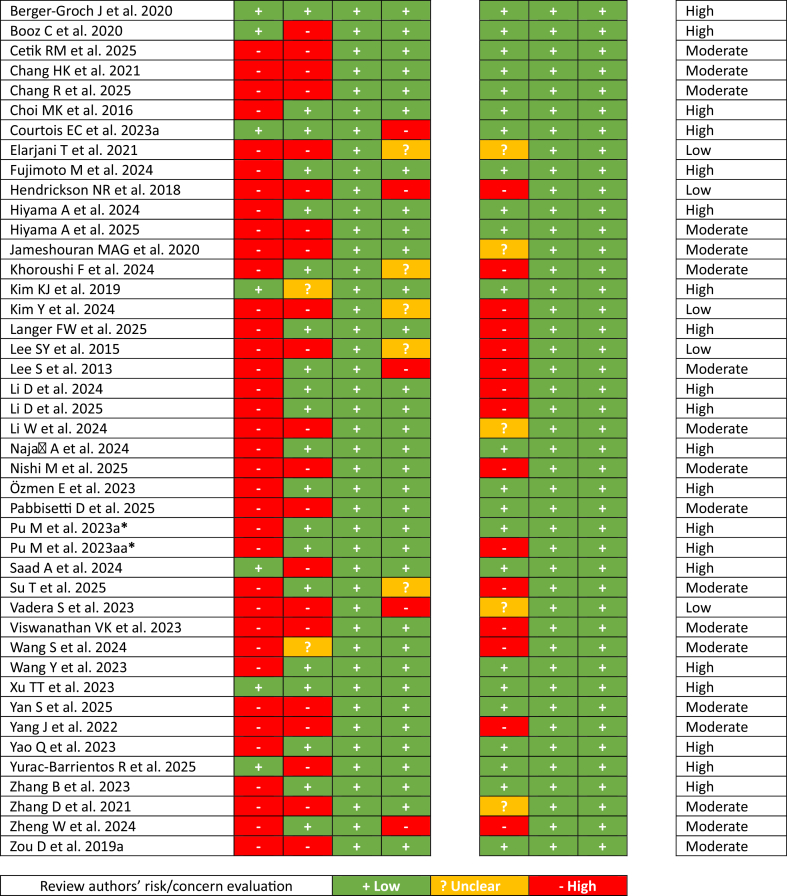
Overview of risk-of-bias and applicability concern assessment, according to the domains of the QUADAS-2 tool, presented as review authors’ judgments about each domain for each included study. QUADAS-2 results were primarily interpreted at the domain level for risk of bias and applicability. The high/moderate/low quality classification was an adapted overall categorization and should not be interpreted as an original QUADAS-2 summary score. *Identical author-publication year combination, but separate articles.

### 3.3 Correlation outcomes

#### 3.3.1 Correlation between HU and lumbar T-score

Sixteen studies (n = 3288) reported correlations between vertebral HU values and lumbar T-scores (Hiyama et al., 2024; Kim et al., 2024; Yang et al., 2022; Zhang et al., 2021; Elarjani et al., 2021; Kim et al., 2019; Zou et al., 2019; Choi et al., 2016; Xu et al., 2023; Pu et al., 2023b; Lee et al., 2015; Lee et al., 2013; Vadera et al., 2023; Nishi et al., 2025; Yurac-Barrientos et al., 2025; Cetik et al., 2025). Pooled Pearson's correlation coefficients (*r*) ranged from 0.551 (95% CI: 0.406-0.669) at L1-L4 to 0.617 (95% CI: 0.536-0.686) at L2, whereas this ranged from 0.629 (95% CI: 0.497-0.732) at L1 to 0.741 (95% CI: 0.578-0.847) at L1-L4 for pooled Spearman's rank correlation coefficients (*ρ*). Heterogeneity was high across all analyses (Table 2).

**Table 2:** Pooled correlation coefficients for different CT-based Hounsfield units measurements and DEXA T-scores, stratified by correlation type (Pearson vs. Spearman).

Reference standard	CT-based HU-measurement	Pearson or Spearman	Number of studies |n	Pooled correlation coefficient (95% CI)	I^2^	p-heterogeneity
**Lumbar T-score**	L1 HU	Pearson	12 | 2071	0.592 (0.528-0.649)	73.6%	<0.001
	L2 HU	Pearson	9 | 1291	0.617 (0.536-0.686)	73.8%	<0.001
	L3 HU	Pearson	9 | 1291	0.593 (0.516-0.660)	69.8%	<0.001
	L4 HU	Pearson	9 | 1329	0.572 (0.480-0.651)	77.5%	<0.001
	L1-L4 HU	Pearson	4 | 450	0.551 (0.406-0.669)	70.9%	0.01
	L1 HU	Spearman	3 | 999	0.629 (0.497-0.732)	77.5%	0.01
	L2 HU	Spearman	2 | 160	0.725 (0.557-0.835)	86.5%	0.01
	L3 HU	Spearman	2 | 160	0.723 (0.569-0.827)	80.7%	0.02
	L4 HU	Spearman	2 | 160	0.653 (0.445-0.794)	85.7%	0.01
	L1-L4 HU	Spearman	2 | 160	0.741 (0.578-0.847)	68.3%	0.08
**Femoral neck T-score**	L1 HU	Pearson	2 | 225	0.371 (0.165-0.545)	0.0%	0.67
	L2 HU	Pearson	2 | 225	0.482 (0.369-0.581)	0.0%	0.64
	L3 HU	Pearson	2 | 225	0.402 (0.166-0.595)	0.0%	0.61
	L4 HU	Pearson	2 | 263	0.395 (0.128-0.609)	0.0%	0.32
	L1-L4 HU	Pearson	1 | 180	0.393 (0.143-0.596)	NA	NA
	L1 HU	Spearman	2 | 160	0.638 (0.482-0.755)	73.3%	0.05
	L2 HU	Spearman	2 | 160	0.646 (0.541-0.730)	46.2%	0.17
	L3 HU	Spearman	2 | 160	0.631 (0.447-0.764)	79.5%	0.03
	L4 HU	Spearman	2 | 160	0.624 (0.411-0.772)	81.7%	0.02
	L1-L4 HU	Spearman	2 | 160	0.675 (0.532-0.781)	49.2%	0.16
**Total hip T-score**	L1 HU	Pearson	1 | 45	0.466 (0.200-0.668)	NA	NA
	L2 HU	Pearson	1 | 45	0.584 (0.351-0.749)	NA	NA
	L3 HU	Pearson	1 | 45	0.524 (0.272-0.709)	NA	NA
	L4 HU	Pearson	2 | 263	0.491 (0.181-0.712)	78.6%	0.03
**Lowest T-score**	L1 HU	Pearson	1 | 191	0.532 (0.330-0.687)	NA	NA
	L2 HU	Pearson	1 | 191	0.570 (0.327-0.742)	NA	NA
	L3 HU	Pearson	1 | 191	0.563 (0.136-0.814)	NA	NA
	L4 HU	Pearson	1 | 191	0.573 (0.139-0.822)	NA	NA
	L1-L4 HU	Pearson	1 | 212	0.630 (0.292-0.828)	NA	NA
	L1 HU	Spearman	3 | 650	0.613 (0.507-0.701)	57.8%	0.09
	L2 HU	Spearman	2 | 160	0.701 (0.550-0.808)	58.2%	0.12
	L3 HU	Spearman	2 | 160	0.684 (0.430-0.837)	81.1%	0.02
	L4 HU	Spearman	2 | 160	0.655 (0.380-0.824)	81.9%	0.02
	L1-L4 HU	Spearman	3 | 322	0.647 (0.463-0.778)	81.4%	0.01

#### 3.3.2 Correlation HU and femoral neck T-score

Five studies (n = 603) reported correlations between vertebral HU values and femoral neck T-scores (Hiyama et al., 2024; Kim et al., 2019; Pu et al., 2023b; Nishi et al., 2025; Cetik et al., 2025). Pooled Pearson's *r* ranged from 0.371 (95% CI: 0.165-0.545) at L1 to 0.482 (95% CI: 0.369-0.581) at L2, while overall Spearman's *ρ* ranged from 0.624 (95% CI: 0.411-0.772) at L4 to 0.675 (95% CI: 0.532-0.781) at L1-L4 (Table 2).

#### 3.3.3 Correlation HU and total hip T-score

Two studies (n = 263) reported correlations between vertebral HU values and total hip T-scores (Nishi et al., 2025; Cetik et al., 2025). Overall Pearson's *r* ranged from 0.466 (95% CI: 0.200-0.668) at L1 to 0.584 (95% CI: 0.351-0.749) at L2. Spearman's *ρ* and the correlation between the mean lumbar HU value and total hip T-score were not reported (Table 2).

#### 3.3.4 Correlation HU and lowest T-score

Six studies (n = 1216) reported correlations between vertebral HU values and lowest T-scores (Hiyama et al., 2024; Pu et al., 2023b; Chang et al., 2025; Langer et al., 2025; Li et al., 2024b; Hiyama et al., 2025). Pooled Pearson's *r* ranged from 0.532 (95% CI: 0.330-0.687) at L1 to 0.630 (95% CI: 0.292-0.828) at L1-L4, whereas this ranged from 0.613 (95% CI: 0.507-0.701) at L1 to 0.701 (95% CI: 0.550-0.808) at L2 for pooled Spearman's *ρ* (Table 2).

#### 3.3.5 Correlation between VBQ-score and lumbar T-score

Eight studies (n = 865) reported correlations between VBQ-scores and lumbar T-scores (Hiyama et al., 2024; Li et al., 2024a; Özmen et al., 2023; Chang et al., 2021; Pu et al., 2023a; Xu et al., 2023; Pabbisetti et al., 2025; Najafi et al., 2024). Pooled Pearson's *r* ranged from −0.340 (95% CI: −0.453 to −0.216) at L4 to −0.460 (95% CI: −0.594 to −0.302) at L1, whereas the pooled Spearman's *ρ* was −0.166 (95% CI: −0.440 to 0.136) at L1-L4. Spearman's correlations were not evaluated for individual vertebrae (Table 3).

**Table 3:** Pooled correlation coefficients for different vertebral bone quality score measurements and DEXA T-scores, stratified by correlation type (Pearson vs. Spearman).

Reference standard	MRI-based VBQ-score measurement	Pearson or Spearman	Number of studies |n	Pooled correlation coefficient (95% CI)	I^2^	p-heterogeneity
**Lumbar T-score**	L1 VBQ	Pearson	2 | 218	−0.460 (−0.594 to −0.302)	47.4%	0.17
	L2 VBQ	Pearson	2 | 218	−0.442 (−0.575 to −0.285)	44.3%	0.18
	L3 VBQ	Pearson	2 | 218	−0.341 (−0.515 to −0.139)	60.6%	0.11
	L4 VBQ	Pearson	2 | 218	−0.340 (−0.453 to −0.216)	0.0%	0.84
	L1-L4 VBQ	Pearson	7 | 765	−0.408 (−0.496 to −0.312)	55.8%	0.03
	L1-L4 VBQ	Spearman	1 | 100	−0.166 (−0.440 to 0.136)	NA	NA
**Femoral neck T-score**	L1 VBQ	Pearson	3 | 658	−0.465 (−0.523 to −0.402)	0.0%	0.80
	L2 VBQ	Pearson	3 | 658	−0.441 (−0.501 to −0.377)	0.0%	0.79
	L3 VBQ	Pearson	3 | 658	−0.422 (−0.483 to −0.357)	0.0%	0.99
	L4 VBQ	Pearson	3 | 658	−0.411 (−0.473 to −0.346)	0.0%	0.91
	L1-L4 VBQ	Pearson	4 | 388	−0.361 (−0.506 to −0.197)	48.0%	0.12
	L1-L4 VBQ	Spearman	2 | 244	−0.333 (−0.507 to −0.132)	76.6%	0.01
**Total hip T-score**	L1-L4 VBQ	Pearson	2 | 231	−0.265 (−0.386 to −0.136)	0.0%	0.53
	L1-L4 VBQ	Spearman	1 | 144	−0.408 (−0.539 to −0.257)	NA	NA
**Lowest T-score**	L1-L4 VBQ	Pearson	7 | 1148	−0.476 (−0.548 to −0.398)	45.9%	0.07
	L1-L4 VBQ	Spearman	2 | 244	−0.315 (−0.467 to −0.146)	78.3%	0.01

#### 3.3.6 Correlation of the VBQ-score and femoral neck T-score

Seven studies (n = 1133) reported correlations between VBQ-scores and femoral neck T-scores (Hiyama et al., 2024; Li et al., 2024a; Özmen et al., 2023; Zheng et al., 2024; Pu et al., 2023a; Su et al., 2025; Wang et al., 2024). Pooled Pearson's *r* ranged from −0.361 (95% CI: −0.506 to −0.197) at L1-L4 to −0.465 (95% CI: −0.523 to −0.402) at L1, whereas the pooled Spearman's *ρ* was −0.333 (95% CI: −0.507 to −0.132) at L1-L4. Spearman's correlations were not reported for individual vertebrae (Table 3).

#### 3.3.7 Correlation between VBQ-score and total hip T-score

Three studies (n = 375) reported correlations between VBQ-scores and total hip T-scores (Li et al., 2024a; Özmen et al., 2023; Wang et al., 2024). The pooled Pearson's *r* was −0.265 (95% CI: −0.386 to −0.136) at L1-L4, whereas the pooled Spearman's *ρ* was −0.408 (95% CI: −0.539 to −0.257) at L1-L4. Correlation was not evaluated for individual vertebral levels (Table 3).

#### 3.3.8 Correlation between VBQ-score and lowest T-score

Nine studies (n = 1392) reported correlations between VBQ-scores and lowest T-scores (Hiyama et al., 2024; Wang et al., 2023; Saad et al., 2024; Li et al., 2024a; Özmen et al., 2023; Pu et al., 2023a; Li et al., 2025; Wang et al., 2024; Li et al., 2024b). The pooled Pearson's *r* was −0.476 (95% CI: −0.548 to −0.398) at L1-L4, whereas the pooled Spearman's *ρ* was −0.315 (95% CI: −0.467 to −0.146) at L1-L4. Correlation was not evaluated for individual vertebral levels (Table 3).

### 3.4 ROC-analyses outcomes

**Fig. 3:**
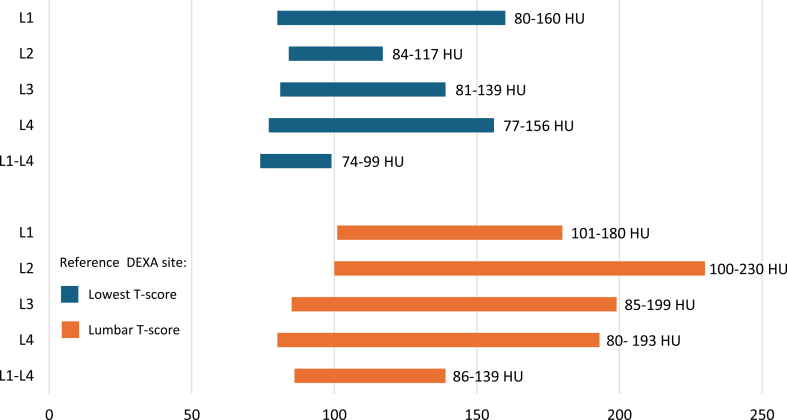
HU cut-off ranges for the diagnosis of osteoporosis, separated by vertebral level and based on lowest T-score and Lumbar T-score as DEXA reference sites.

There were no studies that reported the AUC for vertebral HU values or VBQ-scores to diagnose osteopenia and osteoporosis based on the total hip T-score as the reference standard. The range of reported HU cut-off values for diagnosing osteoporosis was more restricted when based on the lowest T-score, compared to the lumbar T-score (Fig. 3). Across studies, the optimal HU cut-off values, defined as the threshold with the highest combined sensitivity and specificity, varied according to the reference standard and vertebral level assessed. Using the lumbar T-score as the reference standard, the optimal cut-offs were 180 HU at L1 (sensitivity 100.0%, specificity 91.5%), 101 HU at L2 (75.0%, 77.8%), 199 HU at L3 (92.3%, 79.6%), 193 HU at L4 (81.8%, 83.2%), and 88 HU for the average L1–L4 measurement (87.5%, 85.5%). Using the femoral neck T-score as the reference standard, the optimal cut-off for L1–L4 was 99 HU (81.2%, 71.0%). When the lowest T-score was used as the reference standard, the optimal cut-offs were 160 HU at L1 (93.6%, 86.9%), 92 HU at L2 (94.1%, 74.4%), 84 HU at L3 (94.1%, 76.7%), 79 HU at L4 (82.4%, 88.4%), and 92 HU for L1–L4 (94.1%, 74.4%). These findings demonstrate substantial variability in proposed HU thresholds across studies and reference standards. An overview of HU cut-off values to diagnose osteopenia and osteoporosis is provided in Appendix E.

#### 3.4.1 ROC-analysis HU and lumbar T-score

Twelve studies (n = 3023) reported the AUC-based diagnostic discrimination of vertebral HU values using the lumbar T-score as the reference standard (Hendrickson et al., 2018; Hiyama et al., 2024; Zhang et al., 2023; Yang et al., 2022; Booz et al., 2020; Kim et al., 2019; Zou et al., 2019; Fujimoto et al., 2024; Xu et al., 2023; Lee et al., 2015; Vadera et al., 2023; Yurac-Barrientos et al., 2025). For the combined diagnosis of osteopenia and osteoporosis, pooled AUC-values ranged from 0.694 (95% CI: 0.547-0.841) to 0.802 (95% CI: 0.672-0.931), whereas for the diagnosis of osteoporosis alone, pooled AUC-values ranged from 0.807 (95% CI: 0.754-0.860) to 0.853 (95% CI: 0.815-0.891) (Table 4).

**Table 4:** Pooled AUC-values for the diagnosis of osteopenia and osteoporosis using Hounsfield units, based on different DEXA reference standards, stratified by outcome type. No studies used total hip T-score as the reference standard.

Reference standard	CT-based HU-measurement	Outcome	Number of studies |n	Pooled AUC (95% CI)	I^2^	p-heterogeneity
**Lumbar T-score**	L1 HU	Osteopenia + osteoporosis	2 | 983	0.802 (0.672-0.931)	0.0%	0.56
	L1-L4 HU	Osteopenia + osteoporosis	1 | 53	0.694 (0.547-0.841)	NA	NA
	L1 HU	Osteoporosis	7 | 2416	0.834 (0.761-0.907)	94.2%	<0.001
	L2 HU	Osteoporosis	3 | 645	0.853 (0.815-0.891)	0.0%	0.68
	L3 HU	Osteoporosis	3 | 645	0.821 (0.735-0.908)	70.2%	0.04
	L4 HU	Osteoporosis	3 | 645	0.807 (0.754-0.860)	0.0%	0.62
	L1-L4 HU	Osteoporosis	5 | 607	0.845 (0.787-0.902)	58.6%	0.05
**Femoral neck T-score**	L1-L4 HU	Osteoporosis	2 | 280	0.773 (0.736-0.810)	0.0%	0.51

**Lowest T-score**	L1 HU	Osteopenia + osteoporosis	3 | 672	0.819 (0.683-0.954)	35.8%	0.21
	L2 HU	Osteopenia + osteoporosis	1 | 87	0.750 (0.592-0.908)	NA	NA
	L3 HU	Osteopenia + osteoporosis	1 | 87	0.670 (0.512-0.828)	NA	NA
	L4 HU	Osteopenia + osteoporosis	1 | 50	0.730 (0.599-0.861)	NA	NA
	L1-L4 HU	Osteopenia + osteoporosis	1 | 212	0.870 (0.790-0.950)	NA	NA
	L1 HU	Osteoporosis	6 | 1021	0.831 (0.735-0.926)	94.4%	<0.001
	L2 HU	Osteoporosis	3 | 338	0.822 (0.764-0.881)	0.0%	0.68
	L3 HU	Osteoporosis	3 | 338	0.829 (0.780-0.877)	0.0%	0.75
	L4 HU	Osteoporosis	3 | 338	0.859 (0.798-0.920)	27.1%	0.25
	L1-L4 HU	Osteoporosis	3 | 351	0.875 (0.841-0.909)	0.0%	0.37

#### 3.4.2 ROC-analysis HU and femoral neck T-score

Two studies (n = 280) reported the AUC-based diagnostic discrimination of the mean lumbar HU values for diagnosing osteoporosis based on the femoral neck T-score as the reference standard, for which the pooled AUC was 0.773 (95% CI: 0.736-0.810) (Table 4) (Hiyama et al., 2024; Kim et al., 2019).

#### 3.4.3 ROC-analysis HU and lowest T-score

Eleven studies (n = 1626) reported the AUC-based diagnostic discrimination of vertebral HU values using the lowest T-score as the reference standard (Hiyama et al., 2024; Kim et al., 2024; Viswanathan et al., 2023; Yao et al., 2022; Berger-Groch et al., 2020; Pu et al., 2023b; Jameshouran et al., 2020; Chang et al., 2025; Langer et al., 2025; Li et al., 2024b; Khoroushi et al., 2024). For the combined diagnosis of osteopenia and osteoporosis, pooled AUC-values ranged from 0.670 (95% CI: 0.512-0.828) to 0.870 (95% CI: 0.790-0.950), whereas for the diagnosis of osteoporosis alone, pooled AUC-values ranged from 0.822 (95% CI: 0.764-0.881) to 0.875 (95% CI: 0.841-0.909) (Table 4).

#### 3.4.4 ROC-analysis VBQ-score and lumbar T-score

Three studies (n = 314) evaluated the AUC-based diagnostic discrimination of L1-L4 VBQ-scores using the lumbar T-score as the reference standard (Xu et al., 2023; Pabbisetti et al., 2025; Langer et al., 2025). The pooled AUC was 0.612 (95% CI: 0.460-0.764) for the combined diagnosis of osteopenia and osteoporosis and was 0.806 (95% CI: 0.684-0.929) for osteoporosis alone (Table 5).

**Table 5:** Pooled AUC-values for the diagnosis of osteopenia and osteoporosis using the vertebral bone quality score, based on different DEXA reference standards, stratified by outcome type. No studies used total hip T-score as the reference standard.

Reference standard	MRI-based VBQ-score measurement	Outcome	Number of studies |n	Pooled AUC (95% CI)	I^2^	p-heterogeneity
**Lumbar T-score**	L1-L4 VBQ	Osteopenia + osteoporosis	2 | 196	0.612 (0.460-0.764)	91.5%	<0.001
	L1-L4 VBQ	Osteoporosis	3 | 314	0.806 (0.684-0.929)	0.0%	0.47
**Femoral neck T-score**	L1-L4 VBQ	Osteopenia + osteoporosis	1 | 501	0.774 (0.672-0.876)	NA	NA
	L1-L4 VBQ	Osteoporosis	2 | 754	0.692 (0.613-0.772)	68.8%	0.07
**Lowest T-score**	L1-L4 VBQ	Osteopenia + osteoporosis	3 | 583	0.766 (0.675-0.856)	0.0%	0.65
	L1-L4 VBQ	Osteoporosis	2 | 256	0.726 (0.621-0.831)	85.7%	0.01

#### 3.4.5 ROC-analysis VBQ-score and femoral neck T-score

Two studies (n = 754) reported the AUC-based diagnostic discrimination of L1-L4 VBQ-scores using the femoral neck T-score as the reference standard (Su et al., 2025; Yan et al., 2025). For the combined diagnosis of osteopenia and osteoporosis, the pooled AUC value was 0.774 (95% CI: 0.672-0.876), whereas this was 0.692 (95% CI: 0.613-0.772) for osteoporosis alone (Table 5).

#### 3.4.6 ROC-analysis VBQ-score and lowest T-score

Four studies (n = 739) assessed the AUC-based diagnostic discrimination of L1-L4 VBQ-scores using the lowest T-score as the reference standard (Zhang et al., 2023; Wang et al., 2023; Pu et al., 2023a; Li et al., 2024b). The pooled AUC was 0.766 (0.675-0.856) for the combined diagnosis of osteopenia and osteoporosis and was 0.726 (95% CI: 0.621-0.831) for osteoporosis alone (Table 5).

#### 3.4.7 Small study effect

**Fig. 4:**
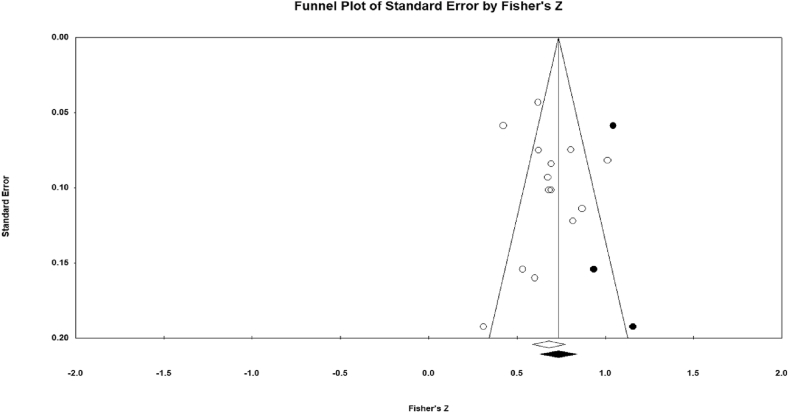
Funnel plot of the standard error against the Fisher's Z value generated for the Pearson's correlation coefficient between L1 HU and lumbar T-score (12 included studies and three imputed potentially missing records). The vertical line represents the logit of the overall pooled correlation coefficient, and the two oblique lines depict the 95% confidence interval for a specific standard error. The trim-and-fill method was conducted to further diagnose potential publication bias. The clear diamond indicates the pooled correlation coefficient of all included studies as represented by the clear dots, while the black diamond indicates the adjusted correlation coefficient after imputing potentially missing records showing a stronger correlation, as represented by the black dots.

Small-study effect was investigated for the Pearson correlation between L1 HU and lumbar T-score, as this was the only outcome with ten or more studies included. There was no evidence of small study effects based on Egger's regression intercept (p = 0.54). Nevertheless, based on visual assessment of asymmetry in the funnel plot, the trim-and-fill method was used to correct for potential publication bias (Fig. 4). Three potentially missing studies were imputed to the right of the pooled point estimate, causing an increase in Pearson's correlation coefficient from 0.592 (95% CI: 0.529-0.648) to 0.626 (95% CI: 0.558-0.686).

#### 3.4.8 Meta-regression of the pooled correlation coefficient by the time interval between CT and DEXA

Meta-regression was performed only for the correlation between L1 HU and lumbar T-score, as fewer than ten studies were available for all other comparisons. Twelve studies including 2071 patients were evaluated, and the pooled Pearson's correlation coefficient showed no significant association with the time interval between CT and DEXA: slope: 0.0000 (95% CI: −0.0009-0.0008; p = 0.96).

#### 3.4.9 Meta-regression of the pooled correlation coefficient by study quality

The same twelve studies were evaluated, and the pooled Pearson's correlation coefficient demonstrated no statistically significant association with study quality (p = 0.10). The regression formula is as follows: Intercept = 0.6666 + (−0.0768 x Low) + (0.1306 x Moderate)

The intercept represents the predicted outcome for studies of high quality, which served as the reference category. Studies of low quality exhibited slightly weaker correlation, while studies of moderate quality demonstrated stronger correlation compared to high-quality studies. However, this difference was not statistically significant (p = 0.10).

#### 3.4.10 Meta-regression of pooled correlation coefficient by percentage of osteoporotic patients

Seven studies (n = 1335) of the total group on which meta-regression could be performed reported the percentage of osteoporotic patients. A significant inverse association was observed between the pooled Pearson's correlation coefficient between L1 HU and lumbar T-score, and the percentage of osteoporotic patients: slope: −0.0066 (95% CI: −0.0106 to −0.0025; p = 0.002). This indicates that every 10 percent increase in the proportion of osteoporotic patients was associated with a decrease of 0.066 (6.6%) in the correlation coefficient.

## 4 Discussion

This meta-analysis aimed to provide insights into the correlation between CT-based HU-measurement, MRI-based VBQ score, and DEXA T-scores, as well as to evaluate the AUC-based diagnostic discrimination of both attenuation measures for detecting osteopenia and osteoporosis across different vertebral levels and according to various DEXA reference sites. HU values appeared to correlate more strongly with DEXA T-scores than VBQ-scores. Moreover, the AUC-based diagnostic discrimination, as reflected by the AUC-values, tended to be higher for HU measurements than for VBQ-scores. Consequently, when available, HU appears to be the better-supported opportunistic marker for low BMD compared to the VBQ-score. However, this should be interpreted in light of indirect comparisons, heterogeneous imaging protocols, and the absence of adjusted head-to-head evidence. Although correlation coefficients were not substantially different across vertebral levels, L2 HU and the mean L1-L4 HU demonstrated the strongest correlations with DEXA T-scores. In addition, correlations between HU and lumbar- and lowest T-scores were the strongest on average, compared to femoral neck- and total hip T-scores. The correlations between the VBQ-score and lumbar- and femoral neck T-scores were highest at L1 and progressively declined from L1 to L4. This can potentially be explained by the increase in lumbar spine fat fraction from L1 to L4 (Lee et al., 2024). Because the VBQ-score estimates the signal intensity based on the vertebral fat content, higher baseline fat in the lower vertebrae reduces the range for detecting differences in bone mineral density, thereby potentially weakening the correlation with DEXA T-scores. Future research could compare the diagnostic performances of L1 VBQ versus L1-L4 VBQ to determine the optimal location to diagnose low BMD. Finally, for HU measurements, Spearman's rank correlation coefficients were consistently higher than Pearson's correlation coefficients across all vertebral levels and DEXA reference sites. In contrast, this pattern was inconclusive for the VBQ-score, likely due to the limited number of studies reporting Spearman's correlations. The higher Spearman's rank correlation coefficients can be attributed to the sensitivity of Pearson's correlation to deviations from normality, including the presence of outliers. In clinical imaging data, extreme HU values may arise from degenerative changes or vertebral fractures, which can weaken Pearson's correlation. In contrast, Spearman's correlation is based on ranked data, making it distribution-free and less affected by outliers, and therefore provides a more reliable estimate of the relationship between HU and DEXA T-scores.

The average L1-L4 HU demonstrated the highest AUC-based diagnostic discrimination for identifying osteoporosis alone, as well as for the combined diagnosis of osteopenia and osteoporosis based on the lowest T-score. However, for the diagnosis of osteoporosis based on the lumbar T-score, L2 HU showed the highest AUC-based diagnostic discrimination. It was not possible to determine which vertebral level is most suitable for the combined diagnosis of osteopenia and osteoporosis based on the lumbar T-score, as HU was only measured at L1 and L1-L4. The AUC-based diagnostic discrimination of the VBQ-score was only assessed for the mean of L1-L4 and not separately for individual vertebrae and showed comparable results across different DEXA measurement sites as reference.

In addition, this meta-analysis demonstrated that the proportion of osteoporotic patients significantly influences the strength of the observed correlation with DEXA T-scores. According to the results of meta-regression, it can be concluded that for every 10% increase in the percentage of osteoporotic patients, the correlation coefficient decreases by 0.066 (6.6%). This finding should be considered when utilizing HU for BMD assessment.

## 5 Strengths and limitations

The authors acknowledge several limitations. First, while efforts were made to standardize the included studies as much as possible, such as excluding patients with systemic pathologies, those receiving BMD-enhancing medications, and studies utilizing intravenous contrast, there was still considerable variability in imaging systems, patient characteristics, and the indications for CT and MRI. These factors may have influenced the results. In addition, we relied on visual inspection to conclude that correlation and AUC-based diagnostic discrimination outcomes were higher for HU than for the VBQ-score, as any test comparing the two point estimates would be subject to confounding bias, due to the unadjusted nature of such comparison. Therefore, the hypothesis that HU outperforms the VBQ-score should be further researched in comparative studies with appropriate adjustment for confounding. Another limitation of this meta-analysis is that technical imaging parameters, such as acquisition and reconstruction settings, were not systematically extracted and analyzed across all included studies. Therefore, their potential contribution to the heterogeneity in HU values and VBQ scores could not be fully assessed. Finally, this meta-analysis did not assess the optimal cut-off values for diagnosing osteopenia and osteoporosis due to inconsistent reporting across studies. The lack of complete contingency data prevented pooling of results and calculation of summary diagnostic accuracy measures. Further research is necessary to address this, as the significant heterogeneity in diagnostic thresholds reported in the literature complicates broader clinical implementation of these opportunistic methods. However, several strengths are worth reporting. This meta-analysis represents, to the best of the authors' knowledge, the first comprehensive comparison of correlation outcomes and AUC-based diagnostic discrimination across different vertebrae and DEXA reference sites. The findings contribute to a better understanding of the utility of CT and MRI in the opportunistic measurement of BMD, particularly focusing on the preferred method and vertebra for assessment.

## 6 Conclusions

In conclusion, HU appeared to be the better-supported opportunistic marker for low BMD compared to the VBQ-score. With respect to HU, there was no substantial variation in correlation outcomes and diagnostic discrimination across different vertebrae. However, on average, HU values from L2 and the mean HU of L1-L4 exhibited marginally stronger correlations with DEXA T-scores and somewhat superior diagnostic discrimination in some analyses. The correlations between the VBQ-score and DEXA T-scores were strongest at L1 and progressively declined from L1 to L4. In clinical practice, these measures may assist in identifying patients at risk of low bone mineral density when dedicated DEXA assessment in unavailable. These findings support HU as a promising opportunistic marker when CT imaging is already available. However, clinical and technical heterogeneity, differences between DXA reference sites and indirect HU-VBQ comparisons restrict clinical interpretability. Standardized acquisition and measurement protocols, threshold-specific validation, and direct comparative studies are still needed before HU can be considered a validated substitute for DXA or before a universally applicable vertebral level, measurement site, or cut-off can be recommended.

## Authors’ contributions

CLAVL and LWFH formulated the research question. CLAVL, RAM, and LWFH contributed to the development of the study design. LWFH and ET screened abstracts, performed full-text screening, and risk-of-bias assessment. LWFH and ET extracted the data. RAM and LWFH performed the data analysis. CLAVL and LWFH interpreted the results of the data analysis. LWFH prepared the first draft of the protocol paper, with contributions and editing from CLAVL, RAM and ET. All authors have read and approved the final manuscript.

## Funding sources

This research did not receive any specific grant from funding agencies in the public, commercial, or not-for-profit sectors.

## Competing interests

CLAVL: Thromboembolic complications in Neurosurgery (Covidien), Fundamentals of radiculopathy (YM Fund/Achmea health insurance and Eurospine), 5yr FU disc prosthesis in cervical spine surgery (CSRS E). Smoking in spondylodesis surgery (Achmea health insurance). Board of CSRS Europe, Netherlands Neurosurgical Society (NVvN) and Eurospine, Advisory Board for Rijndam Rehabilitation centre, faculty for EANS, CSRS, Eurospine spine courses. RAM, ET and LWFH declare that they have no competing interests to disclose.

## Figures and Tables

**Figure:**
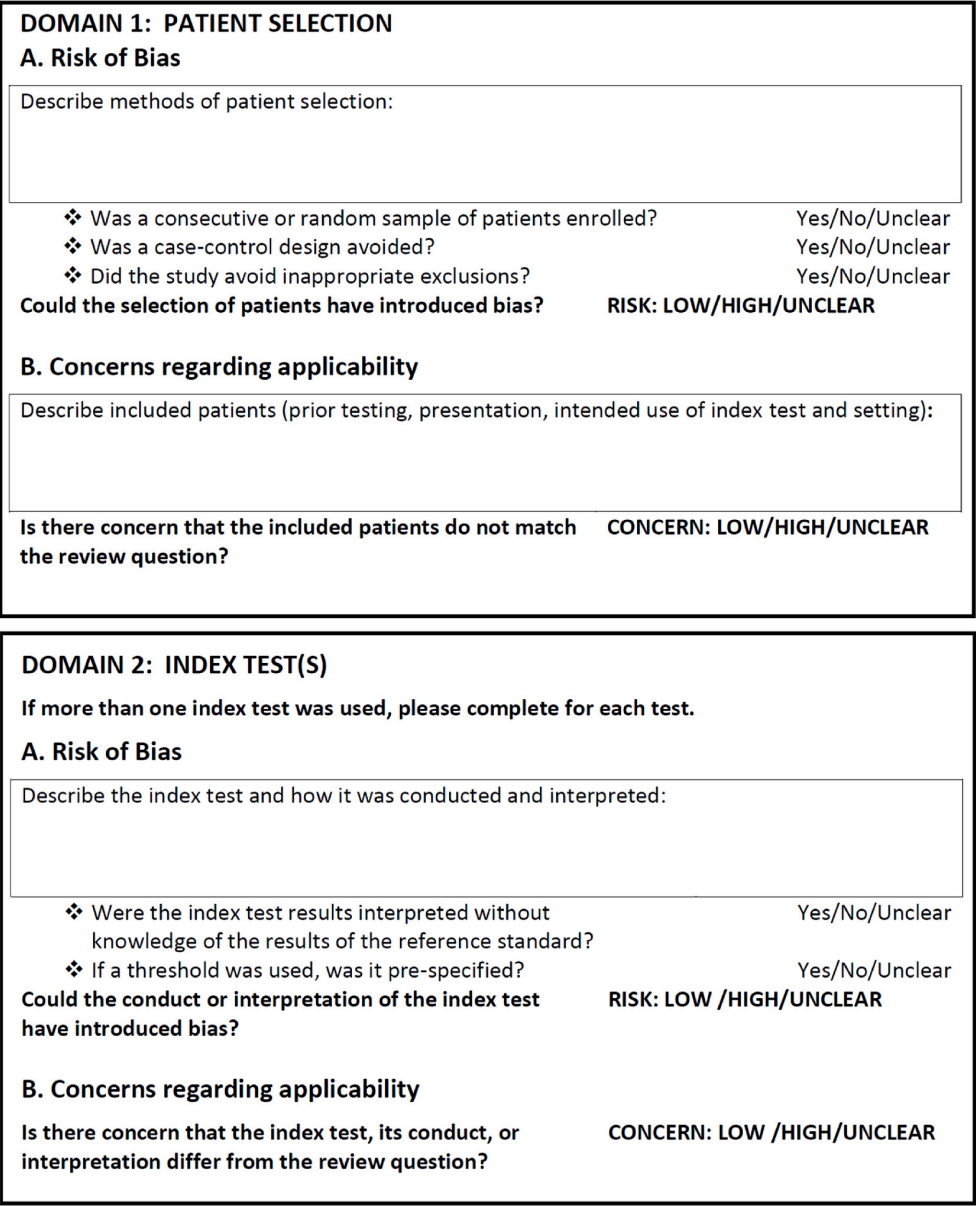


**Figure:**
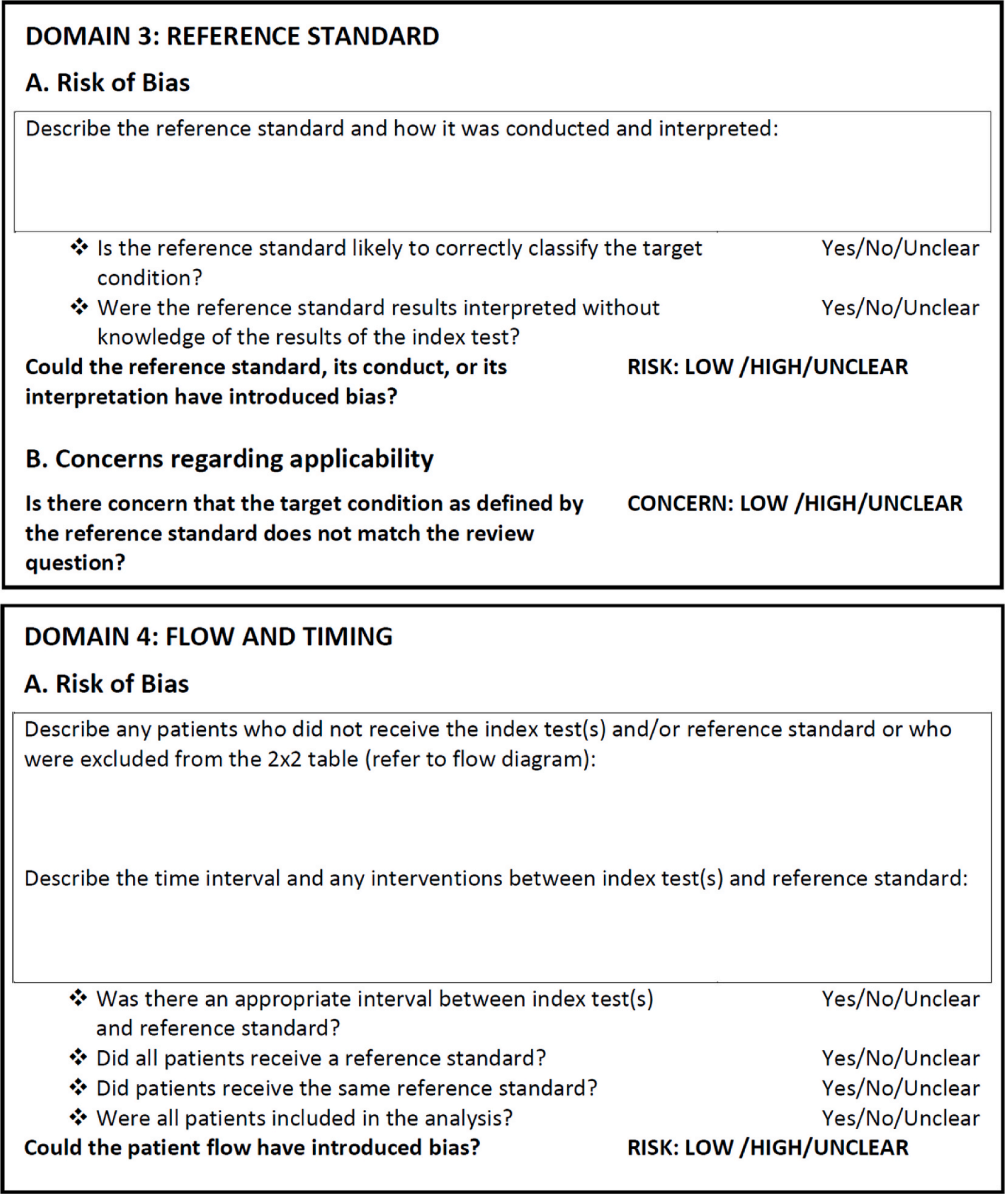


**Table:** 

Author, year, and country of origin	Time between CT/MRI and DEXA (days)	Attenuation value protocol	Hounsfield unit/vertebral bone quality score (SD)	Correlation coefficient | Correlation between	Normal bone density | osteopenia | osteoporosis (%)	Number of patients (mean age)	Study quality and study design
Articles evaluating the correlation between CT and DEXA							
Kim Y, 2024a South-Korea	3.5	Mid-axial HU measurement using ROI	L1: 117.0 (46.4) HU	0.60 |L1-Lumbar T-score	31.0 | 40.0 | 29.0	100 (68.7)	Low Retrospective cohort study
Zhang D, 2021a China	20.0	HU measurement according to ''nine zones'' method, in which nine ROIs are placed on an axial pedicle plane; the mean was used for analysis.	L1: 145.9 (8.7) HU L2: 143.2 (8.6) HU L3: 137.4 (8.3) HU L4: 135.2 (8.2) HU L1-L4: 140.4 (4.2) HU	0.538 |L1-Lumbar T-score 0.435 |L2-Lumbar T-score 0.290 |L3-Lumbar T-score 0.220 |L4-Lumbar T-score 0.349 |L1-L4-Lumbar T-score	-	42 (56.0)	Moderate Prospective cohort study
Elarjani T, 2021a United States of America	365.0	Mid-axial HU measurement using ROI	L1: 170 (66) HU L2: 162 (61) HU L3: 156 (62) HU L4: 160 (64) HU	0.592 |L1-Lumbar T-score 0.482 |L2-Lumbar T-score 0.460 |L3-Lumbar T-score 0.471 |L4-Lumbar T-score	56.0 | 33.0 | 11.0	100 (60.3)	Low Retrospective cohort study
Kim KJ, 2019a South-Korea	91.25	Midcoronal and midsagittal HU-measurement using ROI	- - - - - - - -	0.552 |L1-Lumbar T-score 0.349 |L1-Femoral neck T-score 0.535 |L2-Lumbar T-score 0.469 |L2-Femoral neck T-score 0.542 |L3-Lumbar T-score 0.374 |L3-Femoral neck T-score 0.489 |L1-L3-Lumbar T-score 0.393 |L1-L3-Femoral neck T-score	-	180 (68.1)	High Retrospective cohort study
Zou D, 2019a China Degenerative Non-degenerative	30.4	Mid-axial HU measurement using ROI	L1: 120.2 (39.4) HU L2: 112.1 (38.0) HU L3: 106.1 (37.1) HU L4: 107.0 (41.6) HU L1: 128.1 (35.8) HU L2: 118.1 (35.9) HU L3: 111.5 (35.1) HU L4: 114.7 (37.6) HU	0.667 |L1-Lumbar T-score 0.640 |L2-Lumbar T-score 0.658 |L3-Lumbar T-score 0.667 |L4-Lumbar T-score 0.767 |L1-Lumbar T-score 0.767 |L2-Lumbar T-score 0.717 |L3-Lumbar T-score 0.764 |L4-Lumbar T-score	- -	182 (63.9) 152 (58.5)	Moderate Retrospective cohort study
Yang J, 2022a China	1.0	HU measurement using AI-Rad Companion software and VOI	L1: 131.2 (−) HU	0.6 |L1-Lumbar T-score	22.4 | 49.5 | 28.1 *	839 (59.3) *	Moderate Retrospective cohort study
Choi MK, 2016a South-Korea Degenerative Non-degenerative	91.25	Mid-axial HU measurement using ROI	- - - - - - - - - -	0.300 |L1-Lumbar T-score 0.457 |L2-Lumbar T-score 0.433 |L3-Lumbar T-score 0.447 |L4-Lumbar T-score 0.398 |L1-L4-Lumbar T-score 0.701 |L1-Lumbar T-score 0.709 |L2-Lumbar T-score 0.709 |L3-Lumbar T-score 0.649 |L4-Lumbar T-score 0.398 |L1-L4-Lumbar T-score	- -	30 (67.5) 80 (51.4)	High Retrospective cohort study
Pu M, 2023a China	2.5	Mid-axial, midsagittal, and midcoronal HU measurement using ROI. Mean HU was used for analysis	L1: 114.9 (−) HU L2: 106.4 (−) HU L3: 99.7 (−) HU L4: 102.6 (−) HU L1-L4: 105.9 (−) HU	0.780 |L1-Lumbar T-score 0.734 |L1-Femoral neck T-score 0.738 |L1-Lowest T-score 0.822 |L2-Lumbar T-score 0.721 |L2-Femoral neck T-score 0.767 |L2-Lowest T-score 0.808 |L3-Lumbar T-score 0.738 |L3-Femoral neck T-score 0.777 |L3-Lowest T-score 0.769 |L4-Lumbar T-score 0.736 |L4-Femoral neck T-score 0.757 |L4-Lowest T-score 0.805 |L1-L4-Lumbar T-score 0.741 |L1-L4-Femoral neck T-score 0.769 |L1-L4-Lowest T-score	21.7 | 50.0 | 28.3	60 (66.6)	High Retrospective cohort study
Lee SY, 2015a South Korea	91.25	HU measurement in the coronal plane using a circular ROI in the central part of the vertebral body.	- - - -	0.399 |L1-Lumbar T-score 0.541 |L2-Lumbar T-score 0.613 |L3-Lumbar T-score 0.531 |L4-Lumbar T-score	5.5 | 30.5 | 65.1	292 (73.2)	Low Retrospective cohort study
Lee S, 2013a South Korea	365.0	Mean of axial HU from just below the superior endplate, the center vertebral body, and just above the inferior endplate using ROI	- - - - **	0.673 |L1-Lumbar T-score 0.794 |L2-Lumbar T-score 0.766 |L3-Lumbar T-score 0.713 |L4-Lumbar T-score	-	70 (71.2)	Moderate Retrospective cohort study
Vadera S, 2023a United Kingdom	89.5	HU measurement using ROI in the central portion of the vertebral body over an area of trabecular bone.	L1: 144.2 (−) HU	0.55 |L1-Lumbar T-score	26.5 | 41.0 | 32.5	536 (65.8)	Low Retrospective cohort study
Nishi M, 2025a Japan	91.25	Mid-axial HU measurement using ROI	L4: 115.8 (47.0) HU L5: 120.0 (47.5) HU	0.62 |L4-Lumbar T-score 0.45 |L4-Femoral neck T-score 0.60 |L4-Total hip T-score 0.57 |L5-Lumbar T-score 0.47 |L5-Femoral neck T-score 0.57 |L5-Total hip T-score	32.1 | 52.8 | 15.1	218 (68.3)	Moderate Retrospective cohort study
Yurac-Barrientos R, 2025a Chile	182.5	Mid-axial HU measurement using ROI	L1: 124.6 (40.6) HU	0.6 |L1-Lumbar T-score	36.8 | 52.1 | 11.1	144 (68.4)	High Retrospective cohort study
Chang R, 2025a China	7.0	Mean of axial HU from just below the superior endplate, the center vertebral body, and just above the inferior endplate using ROI	L1: 64.6 (32.1) HU L2: 66.2 (27.8) HU L3: 53.7 (31.6) HU L4: 61.7 (31.4) HU	0.532 |L1-Lowest T-score 0.570 |L2-Lowest T-score 0.563 |L3-Lowest T-score 0.573 |L4-Lowest T-score	- | - | 70.2	191 (70.7)	Moderate Retrospective cohort study
Langer FW, 2025a Brazil	63.5	HU measurement at the pedicle level in the axial plane using ROI	-	0.566 |L1-Lowest T-score	30.1 | 43.8 | 26.1	491 (64.1)	High Retrospective cohort study
Cetik RM, 2025a United States of America	182.5	Mid-axial HU measurement using ROI	L1: 153.8 (67.6) HU L2: 142.2 (60.6) HU L3: 132.2 (64.4) HU L4: 149.4 (115) HU	0.487 |L1-Lumbar T-score 0.411 |L1-Femoral neck T-score 0.466 |L1-Total hip T-score 0.574 |L2-Lumbar T-score 0.530 |L2-Femoral neck T-score 0.584 |L2-Total hip T-score 0.473 |L3-Lumbar T-score 0.447 |L3-Femoral neck T-score 0.524 |L3-Total hip T-score 0.378 |L4-Lumbar T-score 0.306 |L4-Femoral neck T-score 0.317 |L4-Total hip T-score	28.9 | 55.6 | 15.6	45 (63.3)	Moderate Retrospective cohort study
Hiyama A, 2025a Japan	91.25	Mean of axial HU from just below superior endplate, center vertebral body and just above the inferior endplate using ROI	L1-L4: 127.5 (57.4) HU	0.493 |L1-L4-Lowest T-score	26.5 | 37.7 | 35.8	162 (72.3)	Moderate Retrospective cohort study
**Articles evaluating correlation between CT/MRI and DEXA**							
Hiyama A, 2024a Japan	91.25	Mean of axial HU from just below superior endplate, center vertebral body and just above the inferior endplate using ROI VBQ-score calculation by taking the mean signal intensity of L1-L4 vertebral bodies divided by the signal intensity of cerebrospinal fluid at L3 on T1W images	L1: 117.3 (53.2) HU L2: 114.1 (45.7) HU L3: 117.0 (57.5) HU L4: 138.0 (63.7) HU L1-L4: 120.9 (46.1) HU L1-L4: 3.27 (0.70) VBQ	0.504 |L1-Lumbar T-score 0.547 |L1-Femoral neck T-score 0.579 |L1-Lowest T-score 0.610 |L2-Lumbar T-score 0.593 |L2-Femoral neck T-score 0.638 |L2-Lowest T-score 0.630 |L3-Lumbar T-score 0.521 |L3-Femoral neck T-score 0.574 |L3-Lowest T-score 0.520 |L4-Lumbar T-score 0.502 |L4-Femoral neck T-score 0.535 |L4-Lowest T-score 0.673 |L1-L4-Lumbar T-score 0.616 |L1-L4-Femoral neck T-score 0.664 |L1-L4-Lowest T-score −0.166 |L1-L4-Lumbar T-score −0.099 |L1-L4-Femoral neck T-score −0.080 |L1-L4-Lowest T-score	28.0 | 39.0 | 33.0	100 (73.1)	High Retrospective cohort study
Li D, 2024a China	182.5	Mid-axial HU measurement using ROI VBQ-score calculation by taking the mean signal intensity of L1-L4 vertebral bodies divided by the signal intensity of cerebrospinal fluid at L3 on T1W images	L1-L4: 157.2 (52.6) HU L1-L4: 2.73 (0.69) VBQ	0.63 |L1-L4-Lowest T-score −0.56 |L1-L4-Lowest T-score	27.1 | 51.6 | 21.3	212 (59.6)	High Retrospective cohort study
Xu TT, 2023a China	30.4	Mid-axial HU measurement using ROI VBQ-score calculation by taking the mean signal intensity of L1-L4 vertebral bodies divided by the signal intensity of cerebrospinal fluid at L3 on T1W images. In addition, the VBQ-score of the individual segment was calculated by dividing the SI of the single segment by the SI of the corresponding CSF	L1: 133.8 (54.1) HU L2: 125.5 (52.7) HU L3: 123.9 (44.3) HU L4: 115.5 (53.2) HU L1-L4: 123.1 (53.8) HU - - - - L1-L4: 13.24 (2.94) VBQ	0.588 |L1-Lumbar T-score 0.612 |L2-Lumbar T-score 0.519 |L3-Lumbar T-score 0.456 |L4-Lumbar T-score 0.617 |L1-L4-Lumbar T-score −0.386 |L1-Lumbar T-score −0.368 |L2-Lumbar T-score −0.244 |L3-Lumbar T-score −0.351 |L4-Lumbar T-score −0.398 |L1-L4-Lumbar T-score	32.2 | 37.3 | 30.5	118 (59.6)	High Retrospective cohort study
**Articles evaluating correlation between MRI and DEXA**							
Wang Y, 2023c China Male Female	1.0	VBQ-score calculation by taking the median signal intensity of L1-L4 vertebral bodies divided by the signal intensity of cerebrospinal fluid at L3 on T1W images	L1-L4: 3.17 (0.40) VBQ L1-L4: 3.31 (0.50) VBQ	−0.40 |L1-L4-Lowest T-score −0.554 |L1-L4-Lowest T-score	25.0 | - | - 20.1 | - | -	92 (62.0) 179 (62.3)	High Retrospective cohort study
Saad A, 2024a United Kingdom	84.1	VBQ-score calculation by taking the mean signal intensity of L1-L4 vertebral bodies divided by the signal intensity of cerebrospinal fluid at L4 on T1W images	-	−0.474 |L1-L4-Lowest T-score	52.0 | 27.0 | 21.0	100 (63.2)	High Retrospective cohort study
Li W, 2024a China	14.0	VBQ-score calculation by taking the median signal intensity of L1-L4 vertebral bodies divided by the signal intensity of cerebrospinal fluid at L3 on T1W images	-	−0.329 |L1-L4-Lumbar T-score −0.362 |L1-L4 Femoral neck T-score −0.309 |L1-L4-Total hip T-score −0.395 |L1-L4-Lowest T-score	-	101 (58.3)	Moderate Retrospective cohort study
Özmen E, 2023a Turkey	182.5	VBQ-score calculation by taking the median signal intensity of L1-L4 vertebral bodies divided by the signal intensity of cerebrospinal fluid at L3 on T1W images	-	−0.26 |L1-L4-Lumbar T-score −0.20 |L1-L4 Femoral neck T-score −0.23 |L1-L4-Total hip T-score −0.30 |L1-L4-Lowest T-score	33.1 | 48.5 | 18.5	130 (63.4)	High Retrospective cohort study
Pu M, 2023aa China	3.4	VBQ-score calculation by taking the mean signal intensity of L1-L4 vertebral bodies divided by the signal intensity of cerebrospinal fluid at L3 on T1W images. In addition, VBQ-score of individual segment was calculated by dividing SI of the single segment by SI of the corresponding CSF	- - - - -	−0.535 |L1-Lumbar T-score −0.476 |L1-Femoral neck T-score −0.516 |L2-Lumbar T-score −0.387 |L2-Femoral neck T-score −0.437 |L3-Lumbar T-score −0.434 |L3-Femoral neck T-score −0.326 |L4-Lumbar T-score −0.437 |L4-Femoral neck T-score −0.482 |L1-L4-Lumbar T-score −0.469 |L1-L4-Femoral neck T-score −0.524 |L1-L4-Lowest T-score	23.0 | 45.0 | 32.0	100 (66.1)	High Retrospective cohort study
Pabbisetti D, 2025a India Degenerative Non-degenerative	182.5	VBQ-score calculation by taking the mean signal intensity of L1-L4 vertebral bodies divided by the signal intensity of cerebrospinal fluid at L3 on T1W images	L1-L4: 3.33 (−) VBQ L1-L4: 3.16 (−) VBQ	−0.202 |L1-L4-Lumbar T-score −0.502 |L1-L4-Lumbar T-score	- -	58 (70.1) 62 (59.8)	Moderate Retrospective cohort study
Li D, 2025a China	182.5	VBQ-score calculation by taking the mean signal intensity of L1-L4 vertebral bodies divided by the signal intensity of cerebrospinal fluid at L3 on T1W images	-	−0.52 |L1-L4-Lowest T-score	33.3 | 38.9 | 27.8	234 (60.8)	High Retrospective cohort study
Su T, 2025a China	91.25	VBQ-score calculation by taking the mean signal intensity of L1-L4 vertebral bodies divided by the signal intensity of cerebrospinal fluid at L3 on T1W images. In addition, VBQ-score of individual segment was calculated by dividing SI of the single segment by SI of the corresponding CSF	- - - - L1-L4: 3.54 (0.66)	−0.47 |L1-Femoral neck T-score −0.45 |L2-Femoral neck T-score −0.42 |L3-Femoral neck T-score −0.41 |L4-Femoral neck T-score -	26.9 | 51.3 | 21.8	501 (64.8)	Moderate Retrospective cohort study
Najafi A, 2024a Iran	60.8	VBQ-score calculation by taking the mean signal intensity of L1-L4 vertebral bodies divided by the signal intensity of cerebrospinal fluid at L3 on T1W images	L1-L4: 3.22 (0.52) VBQ	−0.415 |L1-L4-Lumbar T-score	38.8 | 38.1 |23.1	134 (67.6)	High Prospective cohort study
Wang S, 2024a China Male Female	60.8	VBQ-score calculation by taking the mean signal intensity of L1-L4 vertebral bodies divided by the signal intensity of cerebrospinal fluid at L3 on T1W images	L1-L4: 2.70 (−) L1-L4: 2.89 (−)	−0.496 |L1-L4-Femoral neck T-score −0.527 |L1-L4-Total hip T-score −0.514 |L1-L4-Lowest T-score −0.419 |L1-L4-Femoral neck T-score −0.338 |L1-L4-Total hip T-score −0.391 |L1-L4-Lowest T-score	38.0 | 40.0 | 22.0 28.7 | 35.1 | 36.2	50 (70.3) 94 (67.3)	Moderate Retrospective cohort study
Zheng W, 2024k China	14.0	VBQ-score calculation by taking the mean signal intensity of L1-L4 vertebral bodies divided by the signal intensity of cerebrospinal fluid at L3 on T1W images. In addition, VBQ-score of individual segment was calculated by dividing the signal intensity of the single segment by the signal intensity of cerebrospinal fluid at L3	L1: 3.73 (0.91) VBQ L2: 3.79 (1.07) VBQ L3: 3.81 (1.10) VBQ L4: 3.78 (1.10) VBQ L1-L4: 3.78 (1.02) VBQ	−0.394 |L1-Femoral neck T-score −0.448 |L2-Femoral neck T-score −0.416 |L3-Femoral neck T-score −0.378 |L4-Femoral neck T-score −0.423 |L1-L4-Femoral neck T-score	- | - | 26.5	57 (69.3)	Moderate Retrospective cohort study
Chang HK, 2021a Taiwan	91.25	VBQ-score calculation by taking the median signal intensity of L1-L4 vertebral bodies divided by the signal intensity of cerebrospinal fluid at a non-specified level on T1W images	L1-L4: 3.42 (0.75) VBQ ***	−0.651 |L1-L4-Lumbar T-score ***	48.4 | 33.9 | 17.7	62 (61.9)	Moderate Prospective cohort study
Courtois EC, 2023aa United States of America	365.0	VBQ-score calculation by taking the median signal intensity of L1-L4 vertebral bodies divided by the signal intensity of cerebrospinal fluid at L3 on T1W images	-	−0.147 |L1-L4-Lumbar T-score	83.3 | 15.7 | 0.9	427 (48.7)	High Retrospective cohort study

Appendix C. Overview of included articles evaluating the correlation between CT/MRI and DEXA. *Demographic data only reported for full cohort (n = 1046), but not for the patients with L1 measurement (n = 839). **HU values are only reported per age subgroup, not for the full cohort. ***Data reported regarding conventional MRI, not synthetic MRI.

**Table:** 

Author and year	Time between CT/MRI and DEXA (days)	Attenuation value protocol	AUC Hounsfield unit/vertebral bone quality score (95% CI or p-value)	Cut off | Sensitivity | Specificity |	Normal bone density | osteopenia | osteoporosis (%)	Number of patients (mean age)	Risk-of-bias
Articles evaluating the AUC-based diagnostic discrimination of CT							
Kim Y, 2024a South Korea	3.5	Mid-axial HU measurement using ROI	L1: 0.78 (0.68-0.87) based on the lowest T-score	L1: 112 HU | 82.8% | 64.8% Detection of osteoporosis	31.0 | 40.0 | 29.0	100 (68.7)	Low Retrospective cohort study
Viswanathan VK, 2023a India	1.0	Mid-axial, midsagittal, and midcoronal HU measurement using ROI. Mean HU was used for analysis	L1: 0.87 (<0.001) L2: 0.75 (0.002) L3: 0.67 (0.033) based on the lowest T-score L1: 0.74 (0.005) L2: 0.83 (<0.001) L3: 0.77 (0.002) L4: 0.74 (0.004) L5: 0.74 (0.004) based on the lowest T-score	L1: 159 HU | 81.6% | 80.0% L2: 162 HU | 80.0% | 71.1% L3: 164 HU | 75.0% | 60.5% Detection of osteopenia + osteoporosis L1: 127 HU | 71.3% | 70.0% L2: 117 HU | 65.5% | 90.0% L3: 139 HU | 75.9% | 80.0% L4: 156 HU | 79.3% | 65.0% L5: 149 HU | 62.1% | 80.0% Detection of osteoporosis	43.7 | 33.4 | 22.9	87 (60.6)	Moderate Prospective cohort study
Yao Q, 2023a China	7.0	HU measurement on axial slices, parallel to inferior and superior endplate and mid-axial using ROI, Mean HU was used for analysis	L1: 0.83 (0.74-0.90) based on Lowest T-score	L1: 149 HU | 73.0% | 86.0% Detection of osteopenia + osteoporosis	39.4 | 40.4 | 20.2	94 (58.8)	High Retrospective cohort study
Booz C, 2020a Germany	30.0	HU measurement using three ROIs on both axial and sagittal planes, positioned in the anterior trabecular bone space	L1-L4: 0.694 (<0.001) based on Lumbar T-score L1-L4: 0.790 (<0.001) based on Lumbar T-score	L1-L4: 188 HU | 82.0% | 53.0% Detection of osteopenia + osteoporosis L1-L4: 139 HU | 65.0% | 93.0% Detection of osteoporosis	28.3 | 28.2 | 43.4	53 (52.2)	High Retrospective cohort study
Berger-Groch J, 2020a Germany	28.0	Mid-axial HU measurement using ROI	L4: 0.73 (0.61-0.85) L5: 0.76 (0.65-0.87) S1: 0.65 (0.57-0.78) based on Lowest T-score	L4: 112 HU | 62.0% | 75.0% L5: 124 HU | 50.0% | 92.0% S1: 163 HU | 50.0% | 79.0% Detection of osteopenia + osteoporosis	52.0 | 40.0 | 8.0	50 (72.3)	High Retrospective cohort study
Kim KJ, 2019a South Korea	91.25	Midcoronal and midsagittal HU-measurement using ROI	L1-L3: 0.781 (0.727-0.836) based on Lumbar T-score L1-L3: 0.767 (0.727-0.836) based on Femoral neck T-score	L1-L3: 95 HU | 82.5% | 66.4% Detection of osteoporosis -	-	180 (68.1)	High Retrospective cohort study
Zou D, 2019a China Non-degenerative	30.4	Mid-axial HU measurement using ROI	L1: 0.860 (0.801-0.918) L2: 0.855 (0.795-0.914) L3: 0.758 (0.682-0.834) L4: 0.811 (0.741-0.882) based on Lumbar T-score	L1: 110 HU | 58.3% | 90.2% L2: 100 HU | 58.2% | 85.6% L3: 85 HU | 40.8% | 84.5% L4: 80 HU | 33.3% | 88.5% Detection of osteoporosis	-	152 (58.5)	Moderate Retrospective cohort study
Yang J, 2022a China	1.0	HU measurement using AI-Rad Companion software and VOI	L1: 0.793 (0.755-0.831) based on Lumbar T-score L1: 0.807 (0.773-0.841) based on Lumbar T-score	L1: 148 HU | 75.5% | 67.7% Detection of osteopenia + osteoporosis L1: 108 HU | 80.7% | 66.5% Detection of osteoporosis	22.4 | 49.5 | 28.1 ***	839 (59.3) ***	Moderate Retrospective cohort study
Pu M, 2023a China	2.5	Mid-axial, midsagittal, and midcoronal HU measurement using ROI. Mean HU was used for analysis	L1: 0.795 (0.67-0.92) L2: 0.858 (0.75-0.96) L3: 0.846 (0.74-0.95) L4: 0.869 (0.76-0.97) L1-L4: 0.859 (0.76-0.96) based on the lowest T-score	L1: 95 HU | 70.6% | 83.7% L2: 92 HU | 94.1% | 74.4% L3: 84 HU | 94.1% | 76.7% L4: 79 HU | 82.4% | 88.4% L1-L4: 92 HU | 94.1% | 74.4% Detection of osteoporosis	21.7 | 50.0 | 28.3	60 (66.6)	High Retrospective cohort study
Lee SY, 2015a South Korea	91.25	HU measurement in the coronal plane using a circular ROI in the central part of the vertebral body.	L1: 0.811 (0.697-0.897) L2: 0.806 (0.692-0.893) L3: 0.810 (0.698-0.894) L4: 0.760 (0.641-0.855) based on Lumbar T-score	L1: 136 HU | 84.6% | 64.3% L2: 101 HU | 75.0% | 77.8% L3: 105 HU | 82.9% | 72.4% L4: 102 HU | 77.5% | 71.4% Detection of osteoporosis	5.5 | 30.5 | 65.1	292 (73.2)	Low Retrospective cohort study
Vadera S, 2023a United Kingdom	89.5	HU measurement using ROI in the central portion of the vertebral body over an area of trabecular bone.	L1: 0.74 (0.69-0.78) based on Lumbar T-score	L1: 169 HU | 90.0% | 36.0% L1: 160 HU | 88.0% | 43.0% L1: 131 HU | 69.0% | 70.0% L1: 110 HU | 48.0% | 85.0% L1: 104 HU | 35.0% | 90.0% Detection of osteoporosis	26.5 | 41.0 | 32.5	536 (65.8)	Low Retrospective cohort study
Nishi M, 2025a Japan Hip osteoarthritis Non-osteoarthritis	91.25	Mid-axial HU measurement using ROI	L4: 0.84 (−) L5: 0.80 (−) based on Lowest T-score L4: 0.82 (−) L5: 0.83 (−) based on Lowest T-score L4: 0.78 (−) L5: 0.78 (−) based on Lowest T-score L4: 0.76 (−) L5: 0.76 (−) based on Lowest T-score	L4: 125 HU | - | - L5: 113 HU | - | - Detection of osteopenia + osteoporosis L4: 119 HU | - | - L5: 101 HU | - | - Detection of osteoporosis L4: 129 HU | - | - L5: 127 HU | - | - Detection of osteopenia + osteoporosis L4: 97 HU | - | - L5: 193 HU | - | - Detection of osteoporosis	- -	116 (−) 102 (−)	Moderate Retrospective cohort study
Yurac-Barrientos R, 2025a Chile	182.5	Mid-axial HU measurement using ROI	L1: 0.81 (0.72-0.9) based on Lumbar T-score L1: 0.810 (0.766-0.900) based on Lumbar T-score	L1: 101 HU | 75.0% | 75.0% L1: 118 HU | 89.0% | 59.0% L1: 80 HU | 40.0% | 90.0% Detection of osteopenia + osteoporosis L1: 126 HU | 79.0% | 73.0% L1: 155 HU | 89.3% | 48.0% L1: 102 HU | 52.0% | 88.7% Detection of osteoporosis	36.8 | 52.1 | 11.1	144 (68.4)	High Retrospective cohort study
Chang R, 2025a China	7.0	Mean of axial HU from just below superior endplate, center vertebral body and just above the inferior endplate using ROI	L1: 0.86 (0.804-0.920) L2: 0.80 (0.713-0.879) L3: 0.83 (0.764-0.889) L4: 0.88 (0.819-0.931) L1-L4: 0.89 (0.839-0.931) based on Lowest T-score	L1: 80 HU | 80.5% | 78.9% L2: 84 HU | 81.5% | 69.1% L3: 81 HU | 84.9% | 67.9% L4: 77 HU | 78.5% | 84.9% L1-L4: 74 HU | 75.4% | 87.7% Detection of osteoporosis	- | - | 70.2	191 (70.7)	Moderate Retrospective cohort study
Langer FW, 2025a Brazil	63.5	HU measurement at the pedicle level in the axial plane using ROI	L1: 0.769 (0.724-0.814) L1: 0.775 (0.728-0.822) based on Lowest T-score	L1: 140 HU | 76.1% | 59.3% Detection of osteopenia + osteoporosis L1: 120 HU | 80.5% | 63.9% Detection of osteoporosis	30.1 | 43.8 | 26.1	491 (64.1)	High Retrospective cohort study
Hendrickson NR, 2018a United States of America	182.5	Mid-axial HU measurement using ROI	L1: 0.825 (0.709-0.901) based on Lumbar T-score *	L1: 110 HU | 48.0% | 90.0% L1: 153 HU | 93.0% | 50.0% Detection of osteoporosis *	-	252 (58.9) *	Low Retrospective cohort study
Fujimoto M, 2024a Japan	6.0	HU measurement using VOI	L1: 0.967 (0.943-0.991) L2: 0.862 (0.807-0.918) L3: 0.888 (0.823-0.953) L4: 0.841 (0.726-0.957) based on Lumbar T-score	L1: 180 HU | 100.0% | 91.5% L2: 230 HU | 96.8% | 63.9% L3: 199 HU | 92.3% | 79.6% L4: 193 HU | 81.8% | 83.2% Detection of osteoporosis	-	201 (72.0) **	High Retrospective cohort study
Jameshouran MAG, 2020a Iran	60.8	HU measurement in axial and coronal plane using ROI over the center of the vertebral body	L1: 0.983 (0.961-1.000) based on Lowest T-score	L1: 160 HU | 93.6% | 86.9% Detection of osteoporosis	25.0 | 41.3 | 33.7	92 (48.8)	Moderate Prospective cohort study
Khoroushi F, 2024a Iran	90.0	HU measurement in the anterior two-thirds of the trabecular part of the vertebral body using ROI. Measurements taken in the midline, two adjacent cuts on the left and two on the right, mean of five measurements was taken	L1: 0.793 (−) based on Lowest T-score	L1: 79 HU | 46.0% | 90.0% L1: 79 HU | 60.0% | 79.0% L1: 79 HU | 69.0% | 74.0% L1: 79 HU | 86.0% | 64.0% L1: 79 HU | 89.0% | 55.0% L1: 79 HU | 92.0% | 43.0% Detection of osteoporosis	16.8 | 22.1 | 61.1	149 (67.7)	Moderate Retrospective cross-sectional study
Hiyama A, 2025a Japan	91.25	Mean of axial HU from just below superior endplate, center vertebral body and just above the inferior endplate using ROI	L1-L4: 0.707 (−) based on Lowest T-score	L1-L4: 100 HU | - | - Detection of osteoporosis	26.5 | 37.7 | 35.8	162 (72.3)	Moderate Retrospective cohort study
**Articles evaluating AUC-based diagnostic discrimination of CT and MRI**							
Hiyama A, 2024a Japan	91.25	Mean of axial HU from just below the superior endplate, the center vertebral body, and just above the inferior endplate using ROI VBQ-score calculation by taking the mean signal intensity of L1-L4 vertebral bodies divided by the signal intensity of cerebrospinal fluid at L3 on T1W images	L1-L4: 0.900 (0.837-0.963) based on Lumbar T-score L1-L4: 0.800 (0.710-0.972) based on Femoral neck T-score L1-L4: 0.826 (0.742-0.909) based on Lowest T-score	L1-L4: 85.6 HU | 85.3% | 100.0% Detection of osteoporosis L1-L4: 98.9 HU | 81.2% | 71.0% Detection of osteoporosis L1-L4: 99 HU | 83.6% | 72.7% Detection of osteoporosis	28.0 | 39.0 | 33.0	100 (73.1)	High Retrospective cohort study
Li D, 2024a China	182.5	Mid-axial HU measurement using ROI VBQ-score calculation by taking the mean signal intensity of L1-L4 vertebral bodies divided by the signal intensity of cerebrospinal fluid at L3 on T1W images	L1-L4: 0.87 (0.79-0.95) based on Lowest T-score L1-L4: 0.74 (0.56-0.81) based on Lowest T-score	- Detection of osteopenia + osteoporosis - Detection of osteopenia + osteoporosis	27.1 | 51.6 | 21.3	212 (59.6)	High Retrospective cohort study
Courtois EC, 2023a United States of America	365.0	Mid-axial HU measurement using ROI VBQ-score calculation by taking the median signal intensity of L1-L4 vertebral bodies divided by the signal intensity of cerebrospinal fluid at L3 on T1W images	L1-L4: 0.832 (−) based on Lumbar T-score L1-L4: 0.557 (−) based on Lumbar T-score	L1-L4: 179 HU | 90.1% | 42.8% Detection of osteopenia + osteoporosis L1-L4: 2.50 VBQ | 54.7% | 51.4% Detection of osteopenia + osteoporosis	83.3 | 15.7 | 0.9 *****	283 (−) 381 (−)	High Retrospective cohort study
Xu TT, 2023a China	30.4	Mid-axial HU measurement using ROI VBQ-score calculation by taking the mean signal intensity of L1-L4 vertebral bodies divided by the signal intensity of cerebrospinal fluid at L3 on T1W images	L1-L4: 0.876 (0.811-0.942) based on Lumbar T-score L1-L4: 0.771 (0.677-0.865) based on Lumbar T-score	L1-L4: 113 HU | 55.6% | 91.5% Detection of osteoporosis L1-L4: 14.32 VBQ | 36.1% | 93.9% Detection of osteoporosis	32.2 | 37.3 | 30.5	118 (59.6)	High Retrospective cohort study
Zhang B, 2023a China	7.0	Mid-axial HU measurement using ROI VBQ-score calculation by taking the mean signal intensity of L1-L4 vertebral bodies divided by the signal intensity of cerebrospinal fluid at L3 on T1W images	L1-L4: 0.848 (<0.001) based on Lumbar T-score L1-L4: 0.641 (0.002) based on the lowest T-score	L1-L4: 88 HU | 87.5% | 85.5% Detection of osteoporosis L1-L4: 3.30 VBQ | 67.1% | 67.5% Detection of osteoporosis	- | - | 48.7	156 (67.5)	High Retrospective cohort study
**Articles evaluating the AUC-based diagnostic discrimination of MRI**							
Yan S, 2025a China	30.4	VBQ-score calculation by taking the median signal intensity of L1-L4 vertebral bodies divided by the signal intensity of cerebrospinal fluid at L3 on T1W images	L1-L4: 0.643 (0.561-0.725) based on Femoral neck T-score	L1-L4: 2.81 VBQ | 70.5% | 56.2% Detection of osteoporosis	25.7 | 50.2 | 24.1	253 (−)	Moderate Retrospective cohort study
Najafi A, 2024a Iran	60.8	VBQ-score calculation by taking the mean signal intensity of L1-L4 vertebral bodies divided by the signal intensity of cerebrospinal fluid at L3 on T1W images	L1-L4: 0.730 (0.647-0.803) based on Lumbar T-score L1-L4: 0.839 (0.765-0.897) based on Lumbar T-score	L1-L4: 3.37 VBQ | 58.5% | 82.7% Detection of osteopenia + osteoporosis L1-L4: 3.38 VBQ | 83.9% | 70.9% Detection of osteoporosis	38.8 | 38.1 |23.1	134 (67.6)	High Prospective cohort study
Özmen E, 2023a Turkey	182.5	VBQ-score calculation by taking the median signal intensity of L1-L4 vertebral bodies divided by the signal intensity of cerebrospinal fluid at L3 on T1W images	L1-L4: 0.667 (−) based on Lowest T-score	L1-L4: 2.70 VBQ | 83.3% | 44.3% L1-L4: 3.00 VBQ | 54.0% | 73.0% L1-L4: 3.50 VBQ | 21.0% | 96.0% Detection of osteoporosis	33.1 | 48.5 | 18.5	130 (63.4)	High Retrospective cohort study
Pabbisetti D, 2025a India Non-degenerative	182.5	VBQ-score calculation by taking the mean signal intensity of L1-L4 vertebral bodies divided by the signal intensity of cerebrospinal fluid at L3 on T1W images	L1-L4: 0.446 (0.298-0.594) based on Lumbar T-score L1-L4: 0.804 (0.640-0.967) based on Lumbar T-score	L1-L4: 2.85 VBQ | 91.7% | 23.5% Detection of osteopenia + osteoporosis L1-L4: 3.25 VBQ | 85.7% | 59.1% L1-L4: 3.55 VBQ | 71.4% | 81.8% Detection of osteoporosis	-	62 (59.8)	Moderate Retrospective cohort study
Pu M, 2023aa China	3.4	VBQ-score calculation by taking the mean signal intensity of L1-L4 vertebral bodies divided by the signal intensity of cerebrospinal fluid at L3 on T1W images. In addition, VBQ-score of the individual segment was calculated by dividing the SI of the single segment by the SI of the corresponding CSF	L1-L4: 0.793 (0.702-0.884) based on the lowest T-score L1-L4: 0.810 (0.721-0.899) based on the lowest T-score	L1-L4: 3.06 VBQ | 63.6% | 87.0% Detection of osteopenia + osteoporosis L1-L4: 3.05 VBQ | 85.7% | 61.8% Detection of osteoporosis	23.0 | 45.0 | 32.0	100 (66.1)	High Retrospective cohort study
Saad A, 2024a United Kingdom	84.1	VBQ-score calculation by taking the mean signal intensity of L1-L4 vertebral bodies divided by the signal intensity of cerebrospinal fluid at L4 on T1W images.	L1-L4: 0.841 (−) based on the lowest T-score L1: 0.839 (0.717-0.960) L2: 0.817 (0.681-0.952) L3: 0.834 (0.713-0.931) L4: 0.808 (0.685-0.931) based on Lowest T-score	L1-L4: 4.14 VBQ | 66.7% | 93.8% L1-L4: 3.00 VBQ | 85.7% | 55.0% L1-L4: 3.50 VBQ | 76.2% | 77.5% L1-L4: 4.00 VBQ | 66.7% | 90.0% L1-L4: 4.50 VBQ | 52.4% | 95.0% L1-L4: 5.00 VBQ | 38.1% | 98.8% Detection of osteoporosis - - - - Detection of osteoporosis	52.0 | 27.0 | 21.0	100 (63.2)	High Retrospective cohort study
Su T, 2025a China	91.25	VBQ-score calculation by taking the mean signal intensity of L1-L4 vertebral bodies divided by the signal intensity of cerebrospinal fluid at L3 on T1W images. In addition, the VBQ-score of the individual segment was calculated by dividing the SI of the single segment by the SI of the corresponding CSF	L1-L4: 0.774 (0.734-0.809) based on the femoral neck T-score L1-L4: 0.726 (0.685-0.765) based on the femoral neck T-score	- Detection of osteopenia + osteoporosis L1-L4: 3.84 VBQ | 58.7% | 78.6% Detection of osteoporosis	26.9 | 51.3 | 21.8	501 (64.8)	Moderate Retrospective cohort study
Wang S, 2024a China Male Female	60.8	VBQ-score calculation by taking the mean signal intensity of L1-L4 vertebral bodies divided by the signal intensity of cerebrospinal fluid at L3 on T1W images	L1-L4: 0.737 (−) based on Lowest T-score L1-L4: 0.865 (−) based on Lowest T-score L1-L4: 0.721 (−) based on Lowest T-score L1-L4: 0.718 (−) based on Lowest T-score	L1-L4: 2.90 VBQ | 54.8% | 89.5% Detection of osteopenia + osteoporosis L1-L4: 2.91 VBQ | 90.9% | 79.5% Detection of osteoporosis L1-L4: 2.82 VBQ | 67.2% | 81.5% Detection of osteopenia + osteoporosis L1-L4: 3.00 VBQ | 76.5% | 73.3% Detection of osteoporosis	38.0 | 40.0 | 22.0 28.7 | 35.1 | 36.2	50 (70.3) 94 (67.3)	Moderate Retrospective cohort study
Wang Y, 2023c China	1.0	VBQ-score calculation by taking the median signal intensity of L1-L4 vertebral bodies divided by the signal intensity of cerebrospinal fluid at L3 on T1W images	L1-L4: 0.77 (<0.001) based on Lowest T-score	L1-L4: 3.13 VBQ | 69.8% | 76.3% Detection of osteopenia + osteoporosis	21.8 | - | -	271 (62.5)	High Retrospective cohort study
Zheng W, 2024k China	14.0	VBQ-score calculation by taking the mean signal intensity of L1-L4 vertebral bodies divided by the signal intensity of cerebrospinal fluid at L3 on T1W images. In addition, VBQ-score of individual segment was calculated by dividing the signal intensity of the single segment by the signal intensity of cerebrospinal fluid at L3	L1: 0.822 (−) L2: 0.822 (−) L3: 0.801 (−) L4: 0.799 (−) L1-L4: 0.816 (−) based on Femoral neck T-score	L1: 3.93 VBQ | 84.6% | 72.7% L2: 4.37 VBQ | 69.2% | 86.6% L3: 4.10 VBQ | 76.9% | 75.0% L4: 4.59 VBQ | 61.5% | 86.6% L1-L4: 4.32 VBQ | 69.2% | 86.4% Detection of osteoporosis	- | - | 26.5	57 (69.3)	Moderate Retrospective cohort study

Appendix D. Overview of included articles evaluating the AUC-based diagnostic discrimination of CT and MRI to diagnose osteopenia and osteoporosis. *Only data of validation cohort is reported.**Median age reported, not mean age. ***Demographic data reported for full cohort (n = 1046), but not for the patients with L1 measurement (n = 839). ****Only data from control group is reported. *****Data regarding osteopenia/osteoporosis reported for full cohort (n = 426), but not for the patients that got CT (n = 283) and MRI (n = 381).

**Table:** 

Diagnosis based on lumbar T-score as reference				
Vertebral level	Cut-off	Sensitivity	Specificity	Diagnosis
L1	101 HU 108 HU 110 HU 131 HU 136 HU 153 HU 180 HU 126 HU 148 HU	0.75 0.807 0.583 0.69 0.846 0.93 1.000 0.79 0.755	0.75 0.665 0.902 0.70 0.643 0.50 0.915 0.73 0.677	Osteoporosis Osteopenia + osteoporosis
L2	100 HU 101 HU 230 HU	0.582 0.750 0.968	0.856 0.778 0.639	Osteoporosis
L3	85 HU 105 HU 199 HU	0.408 0.829 0.923	0.845 0.724 0.796	Osteoporosis
L4	80 HU 102 HU 193 HU	0.333 0.775 0.818	0.885 0.714 0.832	Osteoporosis
L1-L4	86 HU 88 HU 95 HU 113 HU 139 HU 188 HU	0.853 0.875 0.825 0.556 0.65 0.82	1.000 0.855 0.664 0.915 0.93 0.53	Osteoporosis Osteopenia + osteoporosis

**Diagnosis based on femoral neck T-score as reference**				
**Vertebral level**	**Cut-off**	**Sensitivity**	**Specificity**	**Diagnosis**
L1-L4	99 HU	0.812	0.710	Osteoporosis

**Diagnosis based on lowest T-score as reference**				
**Vertebral level**	**Cut-off**	**Sensitivity**	**Specificity**	**Diagnosis**
L1	80 HU 95 HU 112 HU 113 HU 120 HU 127 HU 160 HU 140 HU 149 HU 159 HU	0.805 0.706 0.828 0.86 0.805 0.713 0.936 0.761 0.730 0.816	0.789 0.837 0.648 0.64 0.639 0.70 0.869 0.593 0.860 0.80	Osteoporosis Osteopenia + osteoporosis
L2	84 HU 92 HU 117 HU 162 HU	0.815 0.941 0.655 0.80	0.691 0.744 0.90 0.711	Osteoporosis Osteopenia + osteoporosis
L3	81 HU 84 HU 139 HU 164 HU	0.849 0.941 0.759 0.75	0.679 0.767 0.80 0.605	Osteoporosis Osteopenia + osteoporosis
L4	77 HU 79 HU 156 HU 112 HU	0.785 0.824 0.793 0.62	0.849 0.884 0.65 0.75	Osteoporosis Osteopenia + osteoporosis
L1-L4	74 HU 92 HU 99 HU	0.754 0.941 0.836	0.877 0.744 0.727	Osteoporosis

Appendix E. Overview of HU cut-off values and the corresponding sensitivity and specificity to diagnose osteopenia and osteoporosis at L1-L4 based on different DEXA reference sites.

## List of abbreviations

BMD: bone mineral density (BMD); DEXA: dual-energy X-ray absorptiometry; WHO: World Health Organization; CT: computed tomography; HU: Hounsfield units; MRI: magnetic resonance imaging; VBQ: vertebral bone quality; ROC: receiver operating characteristic; AUC: area under the curve; 95% CI: 95% confidence interval; QUADAS-2: Quality Assessment Tool for Diagnostic Accuracy Studies-2; I^2^: I-squared index; *r*: Pearson's correlation coefficient; *ρ*: Spearman's rank correlation coefficient.

## Appendix A Search strategy

### PubMed:

((("bone quality"[tw] OR "Bone Density"[Mesh] OR "bone density"[tw] OR "bone quality"[title/abstract:∼3] OR "bone density"[title/abstract:∼3] OR "bones quality"[title/abstract:∼3] OR "bones density"[title/abstract:∼3] OR "Bone structure"[tw] OR "Bone structure"[title/abstract:∼3] OR "Bone structures"[title/abstract:∼3] OR "bone strength"[tw]) AND ("Magnetic Resonance Imaging"[majr] OR "Magnetic Resonance Imaging"[ti] OR "MRI"[ti] OR "MR Imaging"[ti] OR "MR Image"[ti] OR "MR Images"[ti] OR "Chemical Shift Imaging"[ti] OR "Magnetic Resonance Image"[ti] OR "Magnetic Resonance Images"[ti] OR "MR Tomographic"[ti] OR "MR Tomography"[ti] OR "NMR Imaging"[ti] OR "NMR Tomography"[ti] OR "NMR Tomographic"[ti] OR "Proton Spin Tomography"[ti] OR "Spin Echo Imaging"[ti] OR "Magnetic Resonance Imaging"[title:∼3] OR "MR Imaging"[title:∼3] OR "MR Image"[title:∼3] OR "MR Images"[title:∼3] OR "Chemical Shift Imaging"[title:∼3] OR "Magnetic Resonance Image"[title:∼3] OR "Magnetic Resonance Images"[title:∼3] OR "MR Tomographic"[title:∼3] OR "MR Tomography"[title:∼3] OR "NMR Imaging"[title:∼3] OR "NMR Tomography"[title:∼3] OR "NMR Tomographic"[title:∼3] OR "Proton Spin Tomography"[title:∼3] OR "Proton Spin Tomographic"[title:∼3] OR "Spin Echo Imaging"[title:∼3] OR "CT"[ti] OR "Tomography, X-Ray Computed"[majr] OR "CAT Scan"[ti] OR "CAT Scans"[ti] OR "Computed Tomography"[ti] OR "Computer Tomography"[ti] OR "Computerised Tomography"[ti] OR "Computerized Tomography"[ti] OR "Electron Beam Tomography"[ti] OR "Computed Tomography"[title:∼3] OR "Computer Tomography"[title:∼3] OR "Computerised Tomography"[title:∼3] OR "Computerized Tomography"[title:∼3] OR "Electron Beam Tomography"[title:∼3] OR "Computed Tomographic"[title:∼3] OR "Computer Tomographic"[title:∼3] OR "Computerised Tomographic"[title:∼3] OR "Computerized Tomographic"[title:∼3] OR "Electron Beam Tomographic"[title:∼3]) AND ("Radiography, Dual-Energy Scanned Projection"[Mesh] OR "dexa"[tw] OR "DXA"[tw] OR "dual energy Xray absorptiometry"[tw] OR "dual energy Xray absorp*"[tw] OR "dual energy X ray absorptiometry"[tw] OR "dual energy X ray absorp*"[tw] OR "dual energy Xray"[tw] OR "dual energy Xray*"[tw] OR "dual energy X ray"[tw] OR "dual energy X rays"[tw] OR "dual energy Xray absorptiometry"[title/abstract:∼3] OR "dual energy X ray absorptiometry"[title/abstract:∼3] OR "dual energy Xray"[title/abstract:∼3] OR "dual energy X ray"[title/abstract:∼3] OR "dual energy X rays"[title/abstract:∼3]) AND ("Lumbar Vertebrae"[Mesh] OR "lumbar"[tw] OR "low back"[tw] OR "lower back"[tw] OR "Hip"[Mesh] OR "Hip Joint"[Mesh:noexp] OR "Hip"[tw] OR "Hips"[tw] OR "Femur"[Mesh] OR "Femur"[tw] OR "vertebral"[tw])) OR (("bone quality"[tw] OR "Bone Density"[Mesh] OR "bone density"[tw] OR "bone quality"[title/abstract:∼3] OR "bone density"[title/abstract:∼3] OR "bones quality"[title/abstract:∼3] OR "bones density"[title/abstract:∼3] OR "Bone structure"[tw] OR "Bone structure"[title/abstract:∼3] OR "Bone structures"[title/abstract:∼3] OR "bone strength"[tw]) AND ("Magnetic Resonance Imaging"[Mesh] OR "Magnetic Resonance Imaging"[tw] OR "MRI"[tw] OR "MR Imaging"[tw] OR "MR Image"[tw] OR "MR Images"[tw] OR "Chemical Shift Imaging"[tw] OR "Magnetic Resonance Image"[tw] OR "Magnetic Resonance Images"[tw] OR "MR Tomographic"[tw] OR "MR Tomography"[tw] OR "NMR Imaging"[tw] OR "NMR Tomography"[tw] OR "NMR Tomographic"[tw] OR "Proton Spin Tomography"[tw] OR "Spin Echo Imaging"[tw] OR "Magnetic Resonance Imaging"[title/abstract:∼3] OR "MR Imaging"[title/abstract:∼3] OR "MR Image"[title/abstract:∼3] OR "MR Images"[title/abstract:∼3] OR "Chemical Shift Imaging"[title/abstract:∼3] OR "Magnetic Resonance Image"[title/abstract:∼3] OR "Magnetic Resonance Images"[title/abstract:∼3] OR "MR Tomographic"[title/abstract:∼3] OR "MR Tomography"[title/abstract:∼3] OR "NMR Imaging"[title/abstract:∼3] OR "NMR Tomography"[title/abstract:∼3] OR "NMR Tomographic"[title/abstract:∼3] OR "Proton Spin Tomography"[title/abstract:∼3] OR "Proton Spin Tomographic"[title/abstract:∼3] OR "Spin Echo Imaging"[title/abstract:∼3] OR "CT"[ti] OR "Tomography, X-Ray Computed"[Mesh] OR "CAT Scan"[tw] OR "CAT Scans"[tw] OR "Computed Tomography"[tw] OR "Computer Tomography"[tw] OR "Computerised Tomography"[tw] OR "Computerized Tomography"[tw] OR "Electron Beam Tomography"[tw] OR "Computed Tomography"[title/abstract:∼3] OR "Computer Tomography"[title/abstract:∼3] OR "Computerised Tomography"[title/abstract:∼3] OR "Computerized Tomography"[title/abstract:∼3] OR "Electron Beam Tomography"[title/abstract:∼3] OR "Computed Tomographic"[title/abstract:∼3] OR "Computer Tomographic"[title/abstract:∼3] OR "Computerised Tomographic"[title/abstract:∼3] OR "Computerized Tomographic"[title/abstract:∼3] OR "Electron Beam Tomographic"[title/abstract:∼3]) AND ("Radiography, Dual-Energy Scanned Projection"[majr] OR "dexa"[ti] OR "DXA"[ti] OR "dual energy Xray absorptiometry"[ti] OR "dual energy Xray absorp*"[ti] OR "dual energy X ray absorptiometry"[ti] OR "dual energy X ray absorp*"[ti] OR "dual energy Xray"[ti] OR "dual energy Xray*"[ti] OR "dual energy X ray"[ti] OR "dual energy X rays"[ti] OR "dual energy Xray absorptiometry"[title:∼3] OR "dual energy X ray absorptiometry"[title:∼3] OR "dual energy Xray"[title:∼3] OR "dual energy X ray"[title:∼3] OR "dual energy X rays"[title:∼3]) AND ("Lumbar Vertebrae"[Mesh] OR "lumbar"[tw] OR "low back"[tw] OR "lower back"[tw] OR "Hip"[Mesh] OR "Hip Joint"[Mesh:noexp] OR "Hip"[tw] OR "Hips"[tw] OR "Femur"[Mesh] OR "Femur"[tw] OR "vertebral"[tw])) OR (("bone quality"[ti] OR "Bone Density"[majr] OR "bone density"[ti] OR "bone quality"[title:∼3] OR "bone density"[title:∼3] OR "bones quality"[title:∼3] OR "bones density"[title:∼3] OR "Bone structure"[ti] OR "Bone structure"[title:∼3] OR "Bone structures"[title:∼3] OR "bone strength"[ti]) AND ("Magnetic Resonance Imaging"[Mesh] OR "Magnetic Resonance Imaging"[tw] OR "MRI"[tw] OR "MR Imaging"[tw] OR "MR Image"[tw] OR "MR Images"[tw] OR "Chemical Shift Imaging"[tw] OR "Magnetic Resonance Image"[tw] OR "Magnetic Resonance Images"[tw] OR "MR Tomographic"[tw] OR "MR Tomography"[tw] OR "NMR Imaging"[tw] OR "NMR Tomography"[tw] OR "NMR Tomographic"[tw] OR "Proton Spin Tomography"[tw] OR "Spin Echo Imaging"[tw] OR "Magnetic Resonance Imaging"[title/abstract:∼3] OR "MR Imaging"[title/abstract:∼3] OR "MR Image"[title/abstract:∼3] OR "MR Images"[title/abstract:∼3] OR "Chemical Shift Imaging"[title/abstract:∼3] OR "Magnetic Resonance Image"[title/abstract:∼3] OR "Magnetic Resonance Images"[title/abstract:∼3] OR "MR Tomographic"[title/abstract:∼3] OR "MR Tomography"[title/abstract:∼3] OR "NMR Imaging"[title/abstract:∼3] OR "NMR Tomography"[title/abstract:∼3] OR "NMR Tomographic"[title/abstract:∼3] OR "Proton Spin Tomography"[title/abstract:∼3] OR "Proton Spin Tomographic"[title/abstract:∼3] OR "Spin Echo Imaging"[title/abstract:∼3] OR "CT"[ti] OR "Tomography, X-Ray Computed"[Mesh] OR "CAT Scan"[tw] OR "CAT Scans"[tw] OR "Computed Tomography"[tw] OR "Computer Tomography"[tw] OR "Computerised Tomography"[tw] OR "Computerized Tomography"[tw] OR "Electron Beam Tomography"[tw] OR "Computed Tomography"[title/abstract:∼3] OR "Computer Tomography"[title/abstract:∼3] OR "Computerised Tomography"[title/abstract:∼3] OR "Computerized Tomography"[title/abstract:∼3] OR "Electron Beam Tomography"[title/abstract:∼3] OR "Computed Tomographic"[title/abstract:∼3] OR "Computer Tomographic"[title/abstract:∼3] OR "Computerised Tomographic"[title/abstract:∼3] OR "Computerized Tomographic"[title/abstract:∼3] OR "Electron Beam Tomographic"[title/abstract:∼3]) AND ("Radiography, Dual-Energy Scanned Projection"[Mesh] OR "dexa"[tw] OR "DXA"[tw] OR "dual energy Xray absorptiometry"[tw] OR "dual energy Xray absorp*"[tw] OR "dual energy X ray absorptiometry"[tw] OR "dual energy X ray absorp*"[tw] OR "dual energy Xray"[tw] OR "dual energy Xray*"[tw] OR "dual energy X ray"[tw] OR "dual energy X rays"[tw] OR "dual energy Xray absorptiometry"[title/abstract:∼3] OR "dual energy X ray absorptiometry"[title/abstract:∼3] OR "dual energy Xray"[title/abstract:∼3] OR "dual energy X ray"[title/abstract:∼3] OR "dual energy X rays"[title/abstract:∼3]) AND ("Lumbar Vertebrae"[majr] OR "lumbar"[ti] OR "low back"[ti] OR "lower back"[ti] OR "Hip"[majr] OR "Hip Joint"[majr:noexp] OR "Hip"[ti] OR "Hips"[ti] OR "Femur"[majr] OR "Femur"[ti] OR "vertebral"[ti]))) NOT ("Animals"[mesh] NOT "Humans"[mesh]) NOT (("Case Reports"[ptyp] OR "case report"[ti] OR "case rep"[all fields]) NOT ("Review"[ptyp] OR "review"[ti] OR "Clinical Study"[ptyp] OR "trial"[ti] OR "RCT"[ti]))

### Embase:

(((exp *"bone quality"/OR "bone quality".ti,ab OR exp *"Bone Density"/OR "bone density".ti,ab OR exp *"Bone Strength"/OR exp *"Bone structure"/OR "Bone structure".ti,ab OR (("bone" ADJ3 ″quality") OR ("bone" ADJ3 ″density") OR ("bones" ADJ3 ″quality") OR ("bones" ADJ3 ″density") OR ("Bone" ADJ3 ″structure") OR ("Bone" ADJ3 ″structures")).ti,ab) AND (exp *"Nuclear Magnetic Resonance Imaging"/OR "Magnetic Resonance Imaging".ti OR "MRI".ti OR "MR Imaging".ti OR "MR Image".ti OR "MR Images".ti OR "Chemical Shift Imaging".ti OR "Magnetic Resonance Image".ti OR "Magnetic Resonance Images".ti OR "MR Tomographic".ti OR "MR Tomography".ti OR "NMR Imaging".ti OR "NMR Tomography".ti OR "NMR Tomographic".ti OR "Proton Spin Tomography".ti OR "Spin Echo Imaging".ti OR (("Magnetic" ADJ3 ″Resonance" ADJ3 ″Imaging") OR ("MR" ADJ3 ″Imaging") OR ("MR" ADJ3 ″Image") OR ("MR" ADJ3 ″Images") OR ("Chemical" ADJ3 ″Shift" ADJ3 ″Imaging") OR ("Magnetic" ADJ3 ″Resonance" ADJ3 ″Image") OR ("Magnetic" ADJ3 ″Resonance" ADJ3 ″Images") OR ("MR" ADJ3 ″Tomographic") OR ("MR" ADJ3 ″Tomography") OR ("NMR" ADJ3 ″Imaging") OR ("NMR" ADJ3 ″Tomography") OR ("NMR" ADJ3 ″Tomographic") OR ("Proton" ADJ3 ″Spin" ADJ3 ″Tomography") OR ("Proton" ADJ3 ″Spin" ADJ3 ″Tomographic") OR ("Spin" ADJ3 ″Echo" ADJ3 ″Imaging")).ti OR exp *"computer assisted tomography"/OR "CT".ti OR "CAT Scan".ti OR "CAT Scans".ti OR "Computed Tomography".ti OR "Computer Tomography".ti OR "Computerised Tomography".ti OR "Computerized Tomography".ti OR "Electron Beam Tomography".ti OR (("Computed" ADJ3 ″Tomography") OR ("Computer" ADJ3 ″Tomography") OR ("Computerised" ADJ3 ″Tomography") OR ("Computerized" ADJ3 ″Tomography") OR ("Electron" ADJ3 ″Beam" ADJ3 ″Tomography") OR ("Computed" ADJ3 ″Tomographic") OR ("Computer" ADJ3 ″Tomographic") OR ("Computerised" ADJ3 ″Tomographic") OR ("Computerized" ADJ3 ″Tomographic") OR ("Electron" ADJ3 ″Beam" ADJ3 ″Tomographic")).ti) AND (exp *"dual energy X ray absorptiometry"/OR "dexa".ti,ab OR "DXA".ti,ab OR "dual energy Xray absorptiometry".ti,ab OR "dual energy Xray absorp*".ti,ab OR "dual energy X ray absorptiometry".ti,ab OR "dual energy X ray absorp*".ti,ab OR "dual energy Xray".ti,ab OR "dual energy Xray*".ti,ab OR "dual energy X ray".ti,ab OR "dual energy X rays".ti,ab OR (("dual" ADJ3 ″energy" ADJ3 ″Xray" ADJ3 ″absorptiometry") OR ("dual" ADJ3 ″energy" ADJ3 ″X" ADJ3 ″ray" ADJ3 ″absorptiometry") OR ("dual" ADJ3 ″energy" ADJ3 ″Xray") OR ("dual" ADJ3 ″energy" ADJ3 ″X" ADJ3 ″ray") OR ("dual" ADJ3 ″energy" ADJ3 ″X" ADJ3 ″rays")).ti,ab) AND (exp *"Lumbar Vertebra"/OR exp *"Lumbar Spine"/OR "lumbar".ti,ab OR "low back".ti,ab OR "lower back".ti,ab OR exp *"Hip"/OR "Hip".ti,ab OR "Hips".ti,ab OR exp *"Femur"/OR "Femur".ti,ab OR "vertebral".ti,ab)) OR ((exp *"bone quality"/OR "bone quality".ti,ab OR exp *"Bone Density"/OR "bone density".ti,ab OR exp *"Bone Strength"/OR exp *"Bone structure"/OR "Bone structure".ti,ab OR (("bone" ADJ3 ″quality") OR ("bone" ADJ3 ″density") OR ("bones" ADJ3 ″quality") OR ("bones" ADJ3 ″density") OR ("Bone" ADJ3 ″structure") OR ("Bone" ADJ3 ″structures")).ti,ab) AND (exp *"Nuclear Magnetic Resonance Imaging"/OR "Magnetic Resonance Imaging".ti,ab OR "MRI".ti,ab OR "MR Imaging".ti,ab OR "MR Image".ti,ab OR "MR Images".ti,ab OR "Chemical Shift Imaging".ti,ab OR "Magnetic Resonance Image".ti,ab OR "Magnetic Resonance Images".ti,ab OR "MR Tomographic".ti,ab OR "MR Tomography".ti,ab OR "NMR Imaging".ti,ab OR "NMR Tomography".ti,ab OR "NMR Tomographic".ti,ab OR "Proton Spin Tomography".ti,ab OR "Spin Echo Imaging".ti,ab OR (("Magnetic" ADJ3 ″Resonance" ADJ3 ″Imaging") OR ("MR" ADJ3 ″Imaging") OR ("MR" ADJ3 ″Image") OR ("MR" ADJ3 ″Images") OR ("Chemical" ADJ3 ″Shift" ADJ3 ″Imaging") OR ("Magnetic" ADJ3 ″Resonance" ADJ3 ″Image") OR ("Magnetic" ADJ3 ″Resonance" ADJ3 ″Images") OR ("MR" ADJ3 ″Tomographic") OR ("MR" ADJ3 ″Tomography") OR ("NMR" ADJ3 ″Imaging") OR ("NMR" ADJ3 ″Tomography") OR ("NMR" ADJ3 ″Tomographic") OR ("Proton" ADJ3 ″Spin" ADJ3 ″Tomography") OR ("Proton" ADJ3 ″Spin" ADJ3 ″Tomographic") OR ("Spin" ADJ3 ″Echo" ADJ3 ″Imaging")).ti,ab OR exp *"computer assisted tomography"/OR "CT".ti,ab OR "CAT Scan".ti,ab OR "CAT Scans".ti,ab OR "Computed Tomography".ti,ab OR "Computer Tomography".ti,ab OR "Computerised Tomography".ti,ab OR "Computerized Tomography".ti,ab OR "Electron Beam Tomography".ti,ab OR (("Computed" ADJ3 "Tomography") OR ("Computer" ADJ3 "Tomography") OR ("Computerised" ADJ3 "Tomography") OR ("Computerized" ADJ3 "Tomography") OR ("Electron" ADJ3 "Beam" ADJ3 "Tomography") OR ("Computed" ADJ3 "Tomographic") OR ("Computer" ADJ3 "Tomographic") OR ("Computerised" ADJ3 "Tomographic") OR ("Computerized" ADJ3 "Tomographic") OR ("Electron" ADJ3 "Beam" ADJ3 "Tomographic")).ti,ab) AND (exp *"dual energy X ray absorptiometry"/ OR "dexa".ti OR "DXA".ti OR "dual energy Xray absorptiometry".ti OR "dual energy Xray absorp*".ti OR "dual energy X ray absorptiometry".ti OR "dual energy X ray absorp*".ti OR "dual energy Xray".ti OR "dual energy Xray*".ti OR "dual energy X ray".ti OR "dual energy X rays".ti OR (("dual" ADJ3 "energy" ADJ3 "Xray" ADJ3 "absorptiometry") OR ("dual" ADJ3 "energy" ADJ3 "X" ADJ3 "ray" ADJ3 "absorptiometry") OR ("dual" ADJ3 "energy" ADJ3 "Xray") OR ("dual" ADJ3 "energy" ADJ3 "X" ADJ3 "ray") OR ("dual" ADJ3 "energy" ADJ3 "X" ADJ3 "rays")).ti) AND (exp *"Lumbar Vertebra"/ OR exp *"Lumbar Spine"/ OR "lumbar".ti,ab OR "low back".ti,ab OR "lower back".ti,ab OR exp *"Hip"/ OR "Hip".ti,ab OR "Hips".ti,ab OR exp *"Femur"/ OR "Femur".ti,ab OR "vertebral".ti,ab)) OR ((exp *"bone quality"/ OR "bone quality".ti OR exp *"Bone Density"/ OR "bone density".ti OR exp *"Bone Strength"/ OR exp *"Bone structure"/ OR "Bone structure".ti OR (("bone" ADJ3 "quality") OR ("bone" ADJ3 "density") OR ("bones" ADJ3 "quality") OR ("bones" ADJ3 "density") OR ("Bone" ADJ3 "structure") OR ("Bone" ADJ3 "structures")).ti) AND (exp *"Nuclear Magnetic Resonance Imaging"/ OR "Magnetic Resonance Imaging".ti,ab OR "MRI".ti,ab OR "MR Imaging".ti,ab OR "MR Image".ti,ab OR "MR Images".ti,ab OR "Chemical Shift Imaging".ti,ab OR "Magnetic Resonance Image".ti,ab OR "Magnetic Resonance Images".ti,ab OR "MR Tomographic".ti,ab OR "MR Tomography".ti,ab OR "NMR Imaging".ti,ab OR "NMR Tomography".ti,ab OR "NMR Tomographic".ti,ab OR "Proton Spin Tomography".ti,ab OR "Spin Echo Imaging".ti,ab OR (("Magnetic" ADJ3 "Resonance" ADJ3 "Imaging") OR ("MR" ADJ3 "Imaging") OR ("MR" ADJ3 "Image") OR ("MR" ADJ3 "Images") OR ("Chemical" ADJ3 "Shift" ADJ3 "Imaging") OR ("Magnetic" ADJ3 "Resonance" ADJ3 "Image") OR ("Magnetic" ADJ3 "Resonance" ADJ3 "Images") OR ("MR" ADJ3 "Tomographic") OR ("MR" ADJ3 "Tomography") OR ("NMR" ADJ3 "Imaging") OR ("NMR" ADJ3 "Tomography") OR ("NMR" ADJ3 "Tomographic") OR ("Proton" ADJ3 "Spin" ADJ3 "Tomography") OR ("Proton" ADJ3 "Spin" ADJ3 "Tomographic") OR ("Spin" ADJ3 "Echo" ADJ3 "Imaging")).ti,ab OR exp *"computer assisted tomography"/ OR "CT".ti,ab OR "CAT Scan".ti,ab OR "CAT Scans".ti,ab OR "Computed Tomography".ti,ab OR "Computer Tomography".ti,ab OR "Computerised Tomography".ti,ab OR "Computerized Tomography".ti,ab OR "Electron Beam Tomography".ti,ab OR (("Computed" ADJ3 "Tomography") OR ("Computer" ADJ3 "Tomography") OR ("Computerised" ADJ3 "Tomography") OR ("Computerized" ADJ3 "Tomography") OR ("Electron" ADJ3 "Beam" ADJ3 "Tomography") OR ("Computed" ADJ3 "Tomographic") OR ("Computer" ADJ3 "Tomographic") OR ("Computerised" ADJ3 "Tomographic") OR ("Computerized" ADJ3 "Tomographic") OR ("Electron" ADJ3 "Beam" ADJ3 "Tomographic")).ti,ab) AND (exp *"dual energy X ray absorptiometry"/ OR "dexa".ti,ab OR "DXA".ti,ab OR "dual energy Xray absorptiometry".ti,ab OR "dual energy Xray absorp*".ti,ab OR "dual energy X ray absorptiometry".ti,ab OR "dual energy X ray absorp*".ti,ab OR "dual energy Xray".ti,ab OR "dual energy Xray*".ti,ab OR "dual energy X ray".ti,ab OR "dual energy X rays".ti,ab OR (("dual" ADJ3 "energy" ADJ3 "Xray" ADJ3 "absorptiometry") OR ("dual" ADJ3 "energy" ADJ3 "X" ADJ3 "ray" ADJ3 "absorptiometry") OR ("dual" ADJ3 "energy" ADJ3 "Xray") OR ("dual" ADJ3 "energy" ADJ3 "X" ADJ3 "ray") OR ("dual" ADJ3 "energy" ADJ3 "X" ADJ3 "rays")).ti,ab) AND (exp *"Lumbar Vertebra"/ OR exp *"Lumbar Spine"/ OR "lumbar".ti OR "low back".ti OR "lower back".ti OR exp *"Hip"/ OR "Hip".ti OR "Hips".ti OR exp *"Femur"/ OR "Femur".ti OR "vertebral".ti))) NOT (exp "Animals"/ NOT exp "Humans"/) NOT (("Case Report"/ OR "case report".ti OR (case AND (report OR reports)).jw) NOT (exp "Review"/ OR "review".ti OR "Clinical Study"/OR exp "Clinical Trial"/OR "trial".ti OR "RCT".ti)) NOT (conference review or conference abstract).pt.

### Web of Science:

(((TI=("bone quality" OR "bone quality" OR "Bone Density" OR "bone density" OR "Bone Strength" OR "Bone structure" OR "Bone structure" OR (("bone" NEAR/3 ″quality") OR ("bone" NEAR/3 ″density") OR ("bones" NEAR/3 ″quality") OR ("bones" NEAR/3 ″density") OR ("Bone" NEAR/3 ″structure") OR ("Bone" NEAR/3 ″structures"))) OR AK=("bone quality" OR "bone quality" OR "Bone Density" OR "bone density" OR "Bone Strength" OR "Bone structure" OR "Bone structure" OR (("bone" NEAR/3 ″quality") OR ("bone" NEAR/3 ″density") OR ("bones" NEAR/3 ″quality") OR ("bones" NEAR/3 ″density") OR ("Bone" NEAR/3 ″structure") OR ("Bone" NEAR/3 ″structures"))) OR AB=("bone quality" OR "bone quality" OR "Bone Density" OR "bone density" OR "Bone Strength" OR "Bone structure" OR "Bone structure" OR (("bone" NEAR/3 ″quality") OR ("bone" NEAR/3 ″density") OR ("bones" NEAR/3 ″quality") OR ("bones" NEAR/3 ″density") OR ("Bone" NEAR/3 ″structure") OR ("Bone" NEAR/3 ″structures")))) AND TI=("Nuclear Magnetic Resonance Imaging" OR "Magnetic Resonance Imaging" OR "MRI" OR "MR Imaging" OR "MR Image" OR "MR Images" OR "Chemical Shift Imaging" OR "Magnetic Resonance Image" OR "Magnetic Resonance Images" OR "MR Tomographic" OR "MR Tomography" OR "NMR Imaging" OR "NMR Tomography" OR "NMR Tomographic" OR "Proton Spin Tomography" OR "Spin Echo Imaging" OR (("Magnetic" NEAR/3 ″Resonance" NEAR/3 ″Imaging") OR ("MR" NEAR/3 ″Imaging") OR ("MR" NEAR/3 ″Image") OR ("MR" NEAR/3 ″Images") OR ("Chemical" NEAR/3 ″Shift" NEAR/3 ″Imaging") OR ("Magnetic" NEAR/3 ″Resonance" NEAR/3 ″Image") OR ("Magnetic" NEAR/3 ″Resonance" NEAR/3 ″Images") OR ("MR" NEAR/3 ″Tomographic") OR ("MR" NEAR/3 ″Tomography") OR ("NMR" NEAR/3 ″Imaging") OR ("NMR" NEAR/3 ″Tomography") OR ("NMR" NEAR/3 ″Tomographic") OR ("Proton" NEAR/3 ″Spin" NEAR/3 ″Tomography") OR ("Proton" NEAR/3 ″Spin" NEAR/3 ″Tomographic") OR ("Spin" NEAR/3 ″Echo" NEAR/3 ″Imaging")) OR "computer assisted tomography" OR "CT" OR "CAT Scan" OR "CAT Scans" OR "Computed Tomography" OR "Computer Tomography" OR "Computerised Tomography" OR "Computerized Tomography" OR "Electron Beam Tomography" OR (("Computed" NEAR/3 ″Tomography") OR ("Computer" NEAR/3 ″Tomography") OR ("Computerised" NEAR/3 ″Tomography") OR ("Computerized" NEAR/3 ″Tomography") OR ("Electron" NEAR/3 ″Beam" NEAR/3 ″Tomography") OR ("Computed" NEAR/3 ″Tomographic") OR ("Computer" NEAR/3 ″Tomographic") OR ("Computerised" NEAR/3 ″Tomographic") OR ("Computerized" NEAR/3 ″Tomographic") OR ("Electron" NEAR/3 ″Beam" NEAR/3 ″Tomographic"))) AND (TI=("dual energy X ray absorptiometry" OR "dexa" OR "DXA" OR "dual energy Xray absorptiometry" OR "dual energy Xray absorp*" OR "dual energy X ray absorptiometry" OR "dual energy X ray absorp*" OR "dual energy Xray" OR "dual energy Xray*" OR "dual energy X ray" OR "dual energy X rays" OR (("dual" NEAR/3 ″energy" NEAR/3 ″Xray" NEAR/3 ″absorptiometry") OR ("dual" NEAR/3 ″energy" NEAR/3 ″X" NEAR/3 ″ray" NEAR/3 ″absorptiometry") OR ("dual" NEAR/3 ″energy" NEAR/3 ″Xray") OR ("dual" NEAR/3 ″energy" NEAR/3 ″X" NEAR/3 ″ray") OR ("dual" NEAR/3 ″energy" NEAR/3 ″X" NEAR/3 ″rays"))) OR AK=("dual energy X ray absorptiometry" OR "dexa" OR "DXA" OR "dual energy Xray absorptiometry" OR "dual energy Xray absorp*" OR "dual energy X ray absorptiometry" OR "dual energy X ray absorp*" OR "dual energy Xray" OR "dual energy Xray*" OR "dual energy X ray" OR "dual energy X rays" OR (("dual" NEAR/3 ″energy" NEAR/3 ″Xray" NEAR/3 ″absorptiometry") OR ("dual" NEAR/3 ″energy" NEAR/3 ″X" NEAR/3 ″ray" NEAR/3 ″absorptiometry") OR ("dual" NEAR/3 ″energy" NEAR/3 ″Xray") OR ("dual" NEAR/3 ″energy" NEAR/3 ″X" NEAR/3 ″ray") OR ("dual" NEAR/3 ″energy" NEAR/3 ″X" NEAR/3 ″rays"))) OR AB=("dual energy X ray absorptiometry" OR "dexa" OR "DXA" OR "dual energy Xray absorptiometry" OR "dual energy Xray absorp*" OR "dual energy X ray absorptiometry" OR "dual energy X ray absorp*" OR "dual energy Xray" OR "dual energy Xray*" OR "dual energy X ray" OR "dual energy X rays" OR (("dual" NEAR/3 ″energy" NEAR/3 ″Xray" NEAR/3 ″absorptiometry") OR ("dual" NEAR/3 ″energy" NEAR/3 ″X" NEAR/3 ″ray" NEAR/3 ″absorptiometry") OR ("dual" NEAR/3 ″energy" NEAR/3 ″Xray") OR ("dual" NEAR/3 ″energy" NEAR/3 ″X" NEAR/3 ″ray") OR ("dual" NEAR/3 ″energy" NEAR/3 ″X" NEAR/3 ″rays")))) AND (TI=("Lumbar Vertebra" OR "Lumbar Spine" OR "lumbar" OR "low back" OR "lower back" OR "Hip" OR "Hip" OR "Hips" OR "Femur" OR "Femur" OR "vertebral") OR AK=("Lumbar Vertebra" OR "Lumbar Spine" OR "lumbar" OR "low back" OR "lower back" OR "Hip" OR "Hip" OR "Hips" OR "Femur" OR "Femur" OR "vertebral") OR AB=("Lumbar Vertebra" OR "Lumbar Spine" OR "lumbar" OR "low back" OR "lower back" OR "Hip" OR "Hip" OR "Hips" OR "Femur" OR "Femur" OR "vertebral"))) OR ((TI=("bone quality" OR "bone quality" OR "Bone Density" OR "bone density" OR "Bone Strength" OR "Bone structure" OR "Bone structure" OR (("bone" NEAR/3 ″quality") OR ("bone" NEAR/3 ″density") OR ("bones" NEAR/3 ″quality") OR ("bones" NEAR/3 ″density") OR ("Bone" NEAR/3 ″structure") OR ("Bone" NEAR/3 ″structures"))) OR AK=("bone quality" OR "bone quality" OR "Bone Density" OR "bone density" OR "Bone Strength" OR "Bone structure" OR "Bone structure" OR (("bone" NEAR/3 ″quality") OR ("bone" NEAR/3 ″density") OR ("bones" NEAR/3 ″quality") OR ("bones" NEAR/3 ″density") OR ("Bone" NEAR/3 ″structure") OR ("Bone" NEAR/3 ″structures"))) OR AB=("bone quality" OR "bone quality" OR "Bone Density" OR "bone density" OR "Bone Strength" OR "Bone structure" OR "Bone structure" OR (("bone" NEAR/3 ″quality") OR ("bone" NEAR/3 ″density") OR ("bones" NEAR/3 ″quality") OR ("bones" NEAR/3 ″density") OR ("Bone" NEAR/3 ″structure") OR ("Bone" NEAR/3 ″structures")))) AND (TI=("Nuclear Magnetic Resonance Imaging" OR "Magnetic Resonance Imaging" OR "MRI" OR "MR Imaging" OR "MR Image" OR "MR Images" OR "Chemical Shift Imaging" OR "Magnetic Resonance Image" OR "Magnetic Resonance Images" OR "MR Tomographic" OR "MR Tomography" OR "NMR Imaging" OR "NMR Tomography" OR "NMR Tomographic" OR "Proton Spin Tomography" OR "Spin Echo Imaging" OR (("Magnetic" NEAR/3 ″Resonance" NEAR/3 ″Imaging") OR ("MR" NEAR/3 ″Imaging") OR ("MR" NEAR/3 ″Image") OR ("MR" NEAR/3 ″Images") OR ("Chemical" NEAR/3 ″Shift" NEAR/3 ″Imaging") OR ("Magnetic" NEAR/3 ″Resonance" NEAR/3 ″Image") OR ("Magnetic" NEAR/3 ″Resonance" NEAR/3 ″Images") OR ("MR" NEAR/3 ″Tomographic") OR ("MR" NEAR/3 ″Tomography") OR ("NMR" NEAR/3 ″Imaging") OR ("NMR" NEAR/3 ″Tomography") OR ("NMR" NEAR/3 ″Tomographic") OR ("Proton" NEAR/3 ″Spin" NEAR/3 ″Tomography") OR ("Proton" NEAR/3 ″Spin" NEAR/3 ″Tomographic") OR ("Spin" NEAR/3 ″Echo" NEAR/3 ″Imaging")) OR "computer assisted tomography" OR "CT" OR "CAT Scan" OR "CAT Scans" OR "Computed Tomography" OR "Computer Tomography" OR "Computerised Tomography" OR "Computerized Tomography" OR "Electron Beam Tomography" OR (("Computed" NEAR/3 ″Tomography") OR ("Computer" NEAR/3 ″Tomography") OR ("Computerised" NEAR/3 ″Tomography") OR ("Computerized" NEAR/3 ″Tomography") OR ("Electron" NEAR/3 ″Beam" NEAR/3 ″Tomography") OR ("Computed" NEAR/3 ″Tomographic") OR ("Computer" NEAR/3 ″Tomographic") OR ("Computerised" NEAR/3 ″Tomographic") OR ("Computerized" NEAR/3 ″Tomographic") OR ("Electron" NEAR/3 ″Beam" NEAR/3 ″Tomographic"))) OR AK=("Nuclear Magnetic Resonance Imaging" OR "Magnetic Resonance Imaging" OR "MRI" OR "MR Imaging" OR "MR Image" OR "MR Images" OR "Chemical Shift Imaging" OR "Magnetic Resonance Image" OR "Magnetic Resonance Images" OR "MR Tomographic" OR "MR Tomography" OR "NMR Imaging" OR "NMR Tomography" OR "NMR Tomographic" OR "Proton Spin Tomography" OR "Spin Echo Imaging" OR (("Magnetic" NEAR/3 ″Resonance" NEAR/3 ″Imaging") OR ("MR" NEAR/3 ″Imaging") OR ("MR" NEAR/3 ″Image") OR ("MR" NEAR/3 ″Images") OR ("Chemical" NEAR/3 ″Shift" NEAR/3 ″Imaging") OR ("Magnetic" NEAR/3 ″Resonance" NEAR/3 ″Image") OR ("Magnetic" NEAR/3 ″Resonance" NEAR/3 ″Images") OR ("MR" NEAR/3 ″Tomographic") OR ("MR" NEAR/3 ″Tomography") OR ("NMR" NEAR/3 ″Imaging") OR ("NMR" NEAR/3 ″Tomography") OR ("NMR" NEAR/3 ″Tomographic") OR ("Proton" NEAR/3 ″Spin" NEAR/3 ″Tomography") OR ("Proton" NEAR/3 ″Spin" NEAR/3 ″Tomographic") OR ("Spin" NEAR/3 ″Echo" NEAR/3 ″Imaging")) OR "computer assisted tomography" OR "CT" OR "CAT Scan" OR "CAT Scans" OR "Computed Tomography" OR "Computer Tomography" OR "Computerised Tomography" OR "Computerized Tomography" OR "Electron Beam Tomography" OR (("Computed" NEAR/3 ″Tomography") OR ("Computer" NEAR/3 ″Tomography") OR ("Computerised" NEAR/3 ″Tomography") OR ("Computerized" NEAR/3 ″Tomography") OR ("Electron" NEAR/3 ″Beam" NEAR/3 ″Tomography") OR ("Computed" NEAR/3 ″Tomographic") OR ("Computer" NEAR/3 ″Tomographic") OR ("Computerised" NEAR/3 ″Tomographic") OR ("Computerized" NEAR/3 ″Tomographic") OR ("Electron" NEAR/3 ″Beam" NEAR/3 ″Tomographic"))) OR AB=("Nuclear Magnetic Resonance Imaging" OR "Magnetic Resonance Imaging" OR "MRI" OR "MR Imaging" OR "MR Image" OR "MR Images" OR "Chemical Shift Imaging" OR "Magnetic Resonance Image" OR "Magnetic Resonance Images" OR "MR Tomographic" OR "MR Tomography" OR "NMR Imaging" OR "NMR Tomography" OR "NMR Tomographic" OR "Proton Spin Tomography" OR "Spin Echo Imaging" OR (("Magnetic" NEAR/3 ″Resonance" NEAR/3 ″Imaging") OR ("MR" NEAR/3 ″Imaging") OR ("MR" NEAR/3 ″Image") OR ("MR" NEAR/3 ″Images") OR ("Chemical" NEAR/3 ″Shift" NEAR/3 ″Imaging") OR ("Magnetic" NEAR/3 ″Resonance" NEAR/3 ″Image") OR ("Magnetic" NEAR/3 ″Resonance" NEAR/3 ″Images") OR ("MR" NEAR/3 ″Tomographic") OR ("MR" NEAR/3 ″Tomography") OR ("NMR" NEAR/3 ″Imaging") OR ("NMR" NEAR/3 ″Tomography") OR ("NMR" NEAR/3 ″Tomographic") OR ("Proton" NEAR/3 ″Spin" NEAR/3 ″Tomography") OR ("Proton" NEAR/3 ″Spin" NEAR/3 ″Tomographic") OR ("Spin" NEAR/3 ″Echo" NEAR/3 ″Imaging")) OR "computer assisted tomography" OR "CT" OR "CAT Scan" OR "CAT Scans" OR "Computed Tomography" OR "Computer Tomography" OR "Computerised Tomography" OR "Computerized Tomography" OR "Electron Beam Tomography" OR (("Computed" NEAR/3 ″Tomography") OR ("Computer" NEAR/3 ″Tomography") OR ("Computerised" NEAR/3 ″Tomography") OR ("Computerized" NEAR/3 ″Tomography") OR ("Electron" NEAR/3 ″Beam" NEAR/3 ″Tomography") OR ("Computed" NEAR/3 ″Tomographic") OR ("Computer" NEAR/3 ″Tomographic") OR ("Computerised" NEAR/3 ″Tomographic") OR ("Computerized" NEAR/3 ″Tomographic") OR ("Electron" NEAR/3 ″Beam" NEAR/3 ″Tomographic")))) AND TI=("dual energy X ray absorptiometry" OR "dexa" OR "DXA" OR "dual energy Xray absorptiometry" OR "dual energy Xray absorp*" OR "dual energy X ray absorptiometry" OR "dual energy X ray absorp*" OR "dual energy Xray" OR "dual energy Xray*" OR "dual energy X ray" OR "dual energy X rays" OR (("dual" NEAR/3 ″energy" NEAR/3 ″Xray" NEAR/3 ″absorptiometry") OR ("dual" NEAR/3 ″energy" NEAR/3 ″X" NEAR/3 ″ray" NEAR/3 ″absorptiometry") OR ("dual" NEAR/3 ″energy" NEAR/3 ″Xray") OR ("dual" NEAR/3 ″energy" NEAR/3 ″X" NEAR/3 ″ray") OR ("dual" NEAR/3 ″energy" NEAR/3 ″X" NEAR/3 ″rays"))) AND (TI=("Lumbar Vertebra" OR "Lumbar Spine" OR "lumbar" OR "low back" OR "lower back" OR "Hip" OR "Hip" OR "Hips" OR "Femur" OR "Femur" OR "vertebral") OR AK=("Lumbar Vertebra" OR "Lumbar Spine" OR "lumbar" OR "low back" OR "lower back" OR "Hip" OR "Hip" OR "Hips" OR "Femur" OR "Femur" OR "vertebral") OR AB=("Lumbar Vertebra" OR "Lumbar Spine" OR "lumbar" OR "low back" OR "lower back" OR "Hip" OR "Hip" OR "Hips" OR "Femur" OR "Femur" OR "vertebral"))) OR (TI=("bone quality" OR "bone quality" OR "Bone Density" OR "bone density" OR "Bone Strength" OR "Bone structure" OR "Bone structure" OR (("bone" NEAR/3 ″quality") OR ("bone" NEAR/3 ″density") OR ("bones" NEAR/3 ″quality") OR ("bones" NEAR/3 ″density") OR ("Bone" NEAR/3 ″structure") OR ("Bone" NEAR/3 ″structures"))) AND (TI=("Nuclear Magnetic Resonance Imaging" OR "Magnetic Resonance Imaging" OR "MRI" OR "MR Imaging" OR "MR Image" OR "MR Images" OR "Chemical Shift Imaging" OR "Magnetic Resonance Image" OR "Magnetic Resonance Images" OR "MR Tomographic" OR "MR Tomography" OR "NMR Imaging" OR "NMR Tomography" OR "NMR Tomographic" OR "Proton Spin Tomography" OR "Spin Echo Imaging" OR (("Magnetic" NEAR/3 ″Resonance" NEAR/3 ″Imaging") OR ("MR" NEAR/3 ″Imaging") OR ("MR" NEAR/3 ″Image") OR ("MR" NEAR/3 ″Images") OR ("Chemical" NEAR/3 ″Shift" NEAR/3 ″Imaging") OR ("Magnetic" NEAR/3 ″Resonance" NEAR/3 ″Image") OR ("Magnetic" NEAR/3 ″Resonance" NEAR/3 ″Images") OR ("MR" NEAR/3 ″Tomographic") OR ("MR" NEAR/3 ″Tomography") OR ("NMR" NEAR/3 ″Imaging") OR ("NMR" NEAR/3 ″Tomography") OR ("NMR" NEAR/3 ″Tomographic") OR ("Proton" NEAR/3 ″Spin" NEAR/3 ″Tomography") OR ("Proton" NEAR/3 ″Spin" NEAR/3 ″Tomographic") OR ("Spin" NEAR/3 ″Echo" NEAR/3 ″Imaging")) OR "computer assisted tomography" OR "CT" OR "CAT Scan" OR "CAT Scans" OR "Computed Tomography" OR "Computer Tomography" OR "Computerised Tomography" OR "Computerized Tomography" OR "Electron Beam Tomography" OR (("Computed" NEAR/3 ″Tomography") OR ("Computer" NEAR/3 ″Tomography") OR ("Computerised" NEAR/3 ″Tomography") OR ("Computerized" NEAR/3 ″Tomography") OR ("Electron" NEAR/3 ″Beam" NEAR/3 ″Tomography") OR ("Computed" NEAR/3 ″Tomographic") OR ("Computer" NEAR/3 ″Tomographic") OR ("Computerised" NEAR/3 ″Tomographic") OR ("Computerized" NEAR/3 ″Tomographic") OR ("Electron" NEAR/3 ″Beam" NEAR/3 ″Tomographic"))) OR AK=("Nuclear Magnetic Resonance Imaging" OR "Magnetic Resonance Imaging" OR "MRI" OR "MR Imaging" OR "MR Image" OR "MR Images" OR "Chemical Shift Imaging" OR "Magnetic Resonance Image" OR "Magnetic Resonance Images" OR "MR Tomographic" OR "MR Tomography" OR "NMR Imaging" OR "NMR Tomography" OR "NMR Tomographic" OR "Proton Spin Tomography" OR "Spin Echo Imaging" OR (("Magnetic" NEAR/3 ″Resonance" NEAR/3 ″Imaging") OR ("MR" NEAR/3 "Imaging") OR ("MR" NEAR/3 "Image") OR ("MR" NEAR/3 "Images") OR ("Chemical" NEAR/3 "Shift" NEAR/3 "Imaging") OR ("Magnetic" NEAR/3 "Resonance" NEAR/3 "Image") OR ("Magnetic" NEAR/3 "Resonance" NEAR/3 "Images") OR ("MR" NEAR/3 "Tomographic") OR ("MR" NEAR/3 "Tomography") OR ("NMR" NEAR/3 "Imaging") OR ("NMR" NEAR/3 "Tomography") OR ("NMR" NEAR/3 "Tomographic") OR ("Proton" NEAR/3 "Spin" NEAR/3 "Tomography") OR ("Proton" NEAR/3 "Spin" NEAR/3 "Tomographic") OR ("Spin" NEAR/3 "Echo" NEAR/3 "Imaging")) OR "computer assisted tomography" OR "CT" OR "CAT Scan" OR "CAT Scans" OR "Computed Tomography" OR "Computer Tomography" OR "Computerised Tomography" OR "Computerized Tomography" OR "Electron Beam Tomography" OR (("Computed" NEAR/3 "Tomography") OR ("Computer" NEAR/3 "Tomography") OR ("Computerised" NEAR/3 "Tomography") OR ("Computerized" NEAR/3 "Tomography") OR ("Electron" NEAR/3 "Beam" NEAR/3 "Tomography") OR ("Computed" NEAR/3 "Tomographic") OR ("Computer" NEAR/3 "Tomographic") OR ("Computerised" NEAR/3 "Tomographic") OR ("Computerized" NEAR/3 "Tomographic") OR ("Electron" NEAR/3 "Beam" NEAR/3 "Tomographic"))) OR AB=("Nuclear Magnetic Resonance Imaging" OR "Magnetic Resonance Imaging" OR "MRI" OR "MR Imaging" OR "MR Image" OR "MR Images" OR "Chemical Shift Imaging" OR "Magnetic Resonance Image" OR "Magnetic Resonance Images" OR "MR Tomographic" OR "MR Tomography" OR "NMR Imaging" OR "NMR Tomography" OR "NMR Tomographic" OR "Proton Spin Tomography" OR "Spin Echo Imaging" OR (("Magnetic" NEAR/3 "Resonance" NEAR/3 "Imaging") OR ("MR" NEAR/3 "Imaging") OR ("MR" NEAR/3 "Image") OR ("MR" NEAR/3 "Images") OR ("Chemical" NEAR/3 "Shift" NEAR/3 "Imaging") OR ("Magnetic" NEAR/3 "Resonance" NEAR/3 "Image") OR ("Magnetic" NEAR/3 "Resonance" NEAR/3 "Images") OR ("MR" NEAR/3 "Tomographic") OR ("MR" NEAR/3 "Tomography") OR ("NMR" NEAR/3 "Imaging") OR ("NMR" NEAR/3 "Tomography") OR ("NMR" NEAR/3 "Tomographic") OR ("Proton" NEAR/3 "Spin" NEAR/3 "Tomography") OR ("Proton" NEAR/3 "Spin" NEAR/3 "Tomographic") OR ("Spin" NEAR/3 "Echo" NEAR/3 "Imaging")) OR "computer assisted tomography" OR "CT" OR "CAT Scan" OR "CAT Scans" OR "Computed Tomography" OR "Computer Tomography" OR "Computerised Tomography" OR "Computerized Tomography" OR "Electron Beam Tomography" OR (("Computed" NEAR/3 "Tomography") OR ("Computer" NEAR/3 "Tomography") OR ("Computerised" NEAR/3 "Tomography") OR ("Computerized" NEAR/3 "Tomography") OR ("Electron" NEAR/3 "Beam" NEAR/3 ″Tomography") OR ("Computed" NEAR/3 ″Tomographic") OR ("Computer" NEAR/3 ″Tomographic") OR ("Computerised" NEAR/3 ″Tomographic") OR ("Computerized" NEAR/3 ″Tomographic") OR ("Electron" NEAR/3 ″Beam" NEAR/3 ″Tomographic")))) AND (TI=("dual energy X ray absorptiometry" OR "dexa" OR "DXA" OR "dual energy Xray absorptiometry" OR "dual energy Xray absorp*" OR "dual energy X ray absorptiometry" OR "dual energy X ray absorp*" OR "dual energy Xray" OR "dual energy Xray*" OR "dual energy X ray" OR "dual energy X rays" OR (("dual" NEAR/3 ″energy" NEAR/3 ″Xray" NEAR/3 ″absorptiometry") OR ("dual" NEAR/3 ″energy" NEAR/3 ″X" NEAR/3 ″ray" NEAR/3 ″absorptiometry") OR ("dual" NEAR/3 ″energy" NEAR/3 ″Xray") OR ("dual" NEAR/3 ″energy" NEAR/3 ″X" NEAR/3 ″ray") OR ("dual" NEAR/3 ″energy" NEAR/3 ″X" NEAR/3 ″rays"))) OR AK=("dual energy X ray absorptiometry" OR "dexa" OR "DXA" OR "dual energy Xray absorptiometry" OR "dual energy Xray absorp*" OR "dual energy X ray absorptiometry" OR "dual energy X ray absorp*" OR "dual energy Xray" OR "dual energy Xray*" OR "dual energy X ray" OR "dual energy X rays" OR (("dual" NEAR/3 ″energy" NEAR/3 ″Xray" NEAR/3 ″absorptiometry") OR ("dual" NEAR/3 ″energy" NEAR/3 ″X" NEAR/3 ″ray" NEAR/3 ″absorptiometry") OR ("dual" NEAR/3 ″energy" NEAR/3 ″Xray") OR ("dual" NEAR/3 ″energy" NEAR/3 ″X" NEAR/3 ″ray") OR ("dual" NEAR/3 ″energy" NEAR/3 ″X" NEAR/3 ″rays"))) OR AB=("dual energy X ray absorptiometry" OR "dexa" OR "DXA" OR "dual energy Xray absorptiometry" OR "dual energy Xray absorp*" OR "dual energy X ray absorptiometry" OR "dual energy X ray absorp*" OR "dual energy Xray" OR "dual energy Xray*" OR "dual energy X ray" OR "dual energy X rays" OR (("dual" NEAR/3 ″energy" NEAR/3 ″Xray" NEAR/3 ″absorptiometry") OR ("dual" NEAR/3 ″energy" NEAR/3 ″X" NEAR/3 ″ray" NEAR/3 ″absorptiometry") OR ("dual" NEAR/3 ″energy" NEAR/3 ″Xray") OR ("dual" NEAR/3 ″energy" NEAR/3 ″X" NEAR/3 ″ray") OR ("dual" NEAR/3 ″energy" NEAR/3 ″X" NEAR/3 ″rays")))) AND TI=("Lumbar Vertebra" OR "Lumbar Spine" OR "lumbar" OR "low back" OR "lower back" OR "Hip" OR "Hip" OR "Hips" OR "Femur" OR "Femur" OR "vertebral"))) **NOT TI=("veterinary" OR "rabbit" OR "rabbits" OR "animal" OR "animals" OR "mouse" OR "mice" OR "rodent" OR "rodents" OR "rat" OR "rats" OR "pig" OR "pigs" OR "porcine" OR "horse" OR "horses" OR "equine" OR "cow" OR "cows" OR "bovine" OR "goat" OR "goats" OR "sheep" OR "ovine" OR "canine" OR "dog" OR "dogs" OR "feline" OR "cat" OR "cats")** NOT (TI=("Case Report") OR AK=("case report")) NOT DT=("meeting abstract")

### Cochrane Library:

((("bone quality" OR "bone density" OR "Bone Strength" OR "Bone structure"):ti,ab,kw AND ("Nuclear Magnetic Resonance Imaging" OR "Magnetic Resonance Imaging" OR "MRI" OR "MR Imaging" OR "MR Image" OR "MR Images" OR "Chemical Shift Imaging" OR "Magnetic Resonance Image" OR "Magnetic Resonance Images" OR "MR Tomographic" OR "MR Tomography" OR "NMR Imaging" OR "NMR Tomography" OR "NMR Tomographic" OR "Proton Spin Tomography" OR "Spin Echo Imaging" OR "computer assisted tomography" OR "CT" OR "CAT Scan" OR "CAT Scans" OR "Computed Tomography" OR "Computer Tomography" OR "Computerised Tomography" OR "Computerized Tomography" OR "Electron Beam Tomography"):ti AND ("dual energy X ray absorptiometry" OR "dexa" OR "DXA" OR "dual energy Xray absorptiometry" OR "dual energy Xray absorp*" OR "dual energy X ray absorptiometry" OR "dual energy X ray absorp*" OR "dual energy Xray" OR "dual energy Xray*" OR "dual energy X ray" OR "dual energy X rays"):ti,ab,kw AND ("Lumbar Vertebra" OR "Lumbar Spine" OR "lumbar" OR "low back" OR "lower back" OR "Hip" OR "Hip" OR "Hips" OR "Femur" OR "Femur" OR "vertebral"):ti,ab,kw) OR (("bone quality" OR "bone density" OR "Bone Strength" OR "Bone structure"):ti,ab,kw AND ("Nuclear Magnetic Resonance Imaging" OR "Magnetic Resonance Imaging" OR "MRI" OR "MR Imaging" OR "MR Image" OR "MR Images" OR "Chemical Shift Imaging" OR "Magnetic Resonance Image" OR "Magnetic Resonance Images" OR "MR Tomographic" OR "MR Tomography" OR "NMR Imaging" OR "NMR Tomography" OR "NMR Tomographic" OR "Proton Spin Tomography" OR "Spin Echo Imaging" OR "computer assisted tomography" OR "CT" OR "CAT Scan" OR "CAT Scans" OR "Computed Tomography" OR "Computer Tomography" OR "Computerised Tomography" OR "Computerized Tomography" OR "Electron Beam Tomography"):ti,ab,kw AND ("dual energy X ray absorptiometry" OR "dexa" OR "DXA" OR "dual energy Xray absorptiometry" OR "dual energy Xray absorp*" OR "dual energy X ray absorptiometry" OR "dual energy X ray absorp*" OR "dual energy Xray" OR "dual energy Xray*" OR "dual energy X ray" OR "dual energy X rays"):ti AND ("Lumbar Vertebra" OR "Lumbar Spine" OR "lumbar" OR "low back" OR "lower back" OR "Hip" OR "Hip" OR "Hips" OR "Femur" OR "Femur" OR "vertebral"):ti,ab,kw) OR (("bone quality" OR "bone density" OR "Bone Strength" OR "Bone structure"):ti AND ("Nuclear Magnetic Resonance Imaging" OR "Magnetic Resonance Imaging" OR "MRI" OR "MR Imaging" OR "MR Image" OR "MR Images" OR "Chemical Shift Imaging" OR "Magnetic Resonance Image" OR "Magnetic Resonance Images" OR "MR Tomographic" OR "MR Tomography" OR "NMR Imaging" OR "NMR Tomography" OR "NMR Tomographic" OR "Proton Spin Tomography" OR "Spin Echo Imaging" OR "computer assisted tomography" OR "CT" OR "CAT Scan" OR "CAT Scans" OR "Computed Tomography" OR "Computer Tomography" OR "Computerised Tomography" OR "Computerized Tomography" OR "Electron Beam Tomography"):ti,ab,kw AND ("dual energy X ray absorptiometry" OR "dexa" OR "DXA" OR "dual energy Xray absorptiometry" OR "dual energy Xray absorp*" OR "dual energy X ray absorptiometry" OR "dual energy X ray absorp*" OR "dual energy Xray" OR "dual energy Xray*" OR "dual energy X ray" OR "dual energy X rays"):ti,ab,kw AND ("Lumbar Vertebra" OR "Lumbar Spine" OR "lumbar" OR "low back" OR "lower back" OR "Hip" OR "Hip" OR "Hips" OR "Femur" OR "Femur" OR "vertebral"):ti))

### CINAHL:

(((TI("bone quality" OR "bone quality" OR "Bone Density" OR "bone density" OR "Bone Strength" OR "Bone structure" OR "Bone structure" OR (("bone" NEAR/3 ″quality") OR ("bone" NEAR/3 ″density") OR ("bones" NEAR/3 ″quality") OR ("bones" NEAR/3 ″density") OR ("Bone" NEAR/3 ″structure") OR ("Bone" NEAR/3 ″structures"))) OR MW("bone quality" OR "bone quality" OR "Bone Density" OR "bone density" OR "Bone Strength" OR "Bone structure" OR "Bone structure" OR (("bone" NEAR/3 ″quality") OR ("bone" NEAR/3 ″density") OR ("bones" NEAR/3 ″quality") OR ("bones" NEAR/3 ″density") OR ("Bone" NEAR/3 ″structure") OR ("Bone" NEAR/3 ″structures"))) OR AB("bone quality" OR "bone quality" OR "Bone Density" OR "bone density" OR "Bone Strength" OR "Bone structure" OR "Bone structure" OR (("bone" NEAR/3 ″quality") OR ("bone" NEAR/3 ″density") OR ("bones" NEAR/3 ″quality") OR ("bones" NEAR/3 ″density") OR ("Bone" NEAR/3 ″structure") OR ("Bone" NEAR/3 ″structures")))) AND TI("Nuclear Magnetic Resonance Imaging" OR "Magnetic Resonance Imaging" OR "MRI" OR "MR Imaging" OR "MR Image" OR "MR Images" OR "Chemical Shift Imaging" OR "Magnetic Resonance Image" OR "Magnetic Resonance Images" OR "MR Tomographic" OR "MR Tomography" OR "NMR Imaging" OR "NMR Tomography" OR "NMR Tomographic" OR "Proton Spin Tomography" OR "Spin Echo Imaging" OR (("Magnetic" NEAR/3 ″Resonance" NEAR/3 ″Imaging") OR ("MR" NEAR/3 ″Imaging") OR ("MR" NEAR/3 ″Image") OR ("MR" NEAR/3 ″Images") OR ("Chemical" NEAR/3 ″Shift" NEAR/3 ″Imaging") OR ("Magnetic" NEAR/3 ″Resonance" NEAR/3 ″Image") OR ("Magnetic" NEAR/3 ″Resonance" NEAR/3 ″Images") OR ("MR" NEAR/3 ″Tomographic") OR ("MR" NEAR/3 ″Tomography") OR ("NMR" NEAR/3 ″Imaging") OR ("NMR" NEAR/3 ″Tomography") OR ("NMR" NEAR/3 ″Tomographic") OR ("Proton" NEAR/3 ″Spin" NEAR/3 ″Tomography") OR ("Proton" NEAR/3 ″Spin" NEAR/3 ″Tomographic") OR ("Spin" NEAR/3 ″Echo" NEAR/3 ″Imaging")) OR "computer assisted tomography" OR "CT" OR "CAT Scan" OR "CAT Scans" OR "Computed Tomography" OR "Computer Tomography" OR "Computerised Tomography" OR "Computerized Tomography" OR "Electron Beam Tomography" OR (("Computed" NEAR/3 ″Tomography") OR ("Computer" NEAR/3 ″Tomography") OR ("Computerised" NEAR/3 ″Tomography") OR ("Computerized" NEAR/3 ″Tomography") OR ("Electron" NEAR/3 ″Beam" NEAR/3 ″Tomography") OR ("Computed" NEAR/3 ″Tomographic") OR ("Computer" NEAR/3 ″Tomographic") OR ("Computerised" NEAR/3 ″Tomographic") OR ("Computerized" NEAR/3 ″Tomographic") OR ("Electron" NEAR/3 ″Beam" NEAR/3 ″Tomographic"))) AND (TI("dual energy X ray absorptiometry" OR "dexa" OR "DXA" OR "dual energy Xray absorptiometry" OR "dual energy Xray absorp*" OR "dual energy X ray absorptiometry" OR "dual energy X ray absorp*" OR "dual energy Xray" OR "dual energy Xray*" OR "dual energy X ray" OR "dual energy X rays" OR (("dual" NEAR/3 ″energy" NEAR/3 ″Xray" NEAR/3 ″absorptiometry") OR ("dual" NEAR/3 ″energy" NEAR/3 ″X" NEAR/3 ″ray" NEAR/3 ″absorptiometry") OR ("dual" NEAR/3 ″energy" NEAR/3 ″Xray") OR ("dual" NEAR/3 ″energy" NEAR/3 ″X" NEAR/3 ″ray") OR ("dual" NEAR/3 ″energy" NEAR/3 ″X" NEAR/3 ″rays"))) OR MW("dual energy X ray absorptiometry" OR "dexa" OR "DXA" OR "dual energy Xray absorptiometry" OR "dual energy Xray absorp*" OR "dual energy X ray absorptiometry" OR "dual energy X ray absorp*" OR "dual energy Xray" OR "dual energy Xray*" OR "dual energy X ray" OR "dual energy X rays" OR (("dual" NEAR/3 ″energy" NEAR/3 ″Xray" NEAR/3 ″absorptiometry") OR ("dual" NEAR/3 ″energy" NEAR/3 ″X" NEAR/3 ″ray" NEAR/3 ″absorptiometry") OR ("dual" NEAR/3 ″energy" NEAR/3 ″Xray") OR ("dual" NEAR/3 ″energy" NEAR/3 ″X" NEAR/3 ″ray") OR ("dual" NEAR/3 ″energy" NEAR/3 ″X" NEAR/3 ″rays"))) OR AB("dual energy X ray absorptiometry" OR "dexa" OR "DXA" OR "dual energy Xray absorptiometry" OR "dual energy Xray absorp*" OR "dual energy X ray absorptiometry" OR "dual energy X ray absorp*" OR "dual energy Xray" OR "dual energy Xray*" OR "dual energy X ray" OR "dual energy X rays" OR (("dual" NEAR/3 ″energy" NEAR/3 ″Xray" NEAR/3 ″absorptiometry") OR ("dual" NEAR/3 ″energy" NEAR/3 ″X" NEAR/3 ″ray" NEAR/3 ″absorptiometry") OR ("dual" NEAR/3 ″energy" NEAR/3 ″Xray") OR ("dual" NEAR/3 ″energy" NEAR/3 ″X" NEAR/3 ″ray") OR ("dual" NEAR/3 ″energy" NEAR/3 ″X" NEAR/3 ″rays")))) AND (TI("Lumbar Vertebra" OR "Lumbar Spine" OR "lumbar" OR "low back" OR "lower back" OR "Hip" OR "Hip" OR "Hips" OR "Femur" OR "Femur" OR "vertebral") OR MW("Lumbar Vertebra" OR "Lumbar Spine" OR "lumbar" OR "low back" OR "lower back" OR "Hip" OR "Hip" OR "Hips" OR "Femur" OR "Femur" OR "vertebral") OR AB("Lumbar Vertebra" OR "Lumbar Spine" OR "lumbar" OR "low back" OR "lower back" OR "Hip" OR "Hip" OR "Hips" OR "Femur" OR "Femur" OR "vertebral"))) OR ((TI("bone quality" OR "bone quality" OR "Bone Density" OR "bone density" OR "Bone Strength" OR "Bone structure" OR "Bone structure" OR (("bone" NEAR/3 ″quality") OR ("bone" NEAR/3 ″density") OR ("bones" NEAR/3 ″quality") OR ("bones" NEAR/3 ″density") OR ("Bone" NEAR/3 ″structure") OR ("Bone" NEAR/3 ″structures"))) OR MW("bone quality" OR "bone quality" OR "Bone Density" OR "bone density" OR "Bone Strength" OR "Bone structure" OR "Bone structure" OR (("bone" NEAR/3 ″quality") OR ("bone" NEAR/3 ″density") OR ("bones" NEAR/3 ″quality") OR ("bones" NEAR/3 ″density") OR ("Bone" NEAR/3 ″structure") OR ("Bone" NEAR/3 ″structures"))) OR AB("bone quality" OR "bone quality" OR "Bone Density" OR "bone density" OR "Bone Strength" OR "Bone structure" OR "Bone structure" OR (("bone" NEAR/3 ″quality") OR ("bone" NEAR/3 ″density") OR ("bones" NEAR/3 ″quality") OR ("bones" NEAR/3 ″density") OR ("Bone" NEAR/3 ″structure") OR ("Bone" NEAR/3 ″structures")))) AND (TI("Nuclear Magnetic Resonance Imaging" OR "Magnetic Resonance Imaging" OR "MRI" OR "MR Imaging" OR "MR Image" OR "MR Images" OR "Chemical Shift Imaging" OR "Magnetic Resonance Image" OR "Magnetic Resonance Images" OR "MR Tomographic" OR "MR Tomography" OR "NMR Imaging" OR "NMR Tomography" OR "NMR Tomographic" OR "Proton Spin Tomography" OR "Spin Echo Imaging" OR (("Magnetic" NEAR/3 ″Resonance" NEAR/3 ″Imaging") OR ("MR" NEAR/3 ″Imaging") OR ("MR" NEAR/3 ″Image") OR ("MR" NEAR/3 ″Images") OR ("Chemical" NEAR/3 ″Shift" NEAR/3 ″Imaging") OR ("Magnetic" NEAR/3 ″Resonance" NEAR/3 ″Image") OR ("Magnetic" NEAR/3 ″Resonance" NEAR/3 ″Images") OR ("MR" NEAR/3 ″Tomographic") OR ("MR" NEAR/3 ″Tomography") OR ("NMR" NEAR/3 ″Imaging") OR ("NMR" NEAR/3 ″Tomography") OR ("NMR" NEAR/3 ″Tomographic") OR ("Proton" NEAR/3 ″Spin" NEAR/3 ″Tomography") OR ("Proton" NEAR/3 ″Spin" NEAR/3 ″Tomographic") OR ("Spin" NEAR/3 ″Echo" NEAR/3 ″Imaging")) OR "computer assisted tomography" OR "CT" OR "CAT Scan" OR "CAT Scans" OR "Computed Tomography" OR "Computer Tomography" OR "Computerised Tomography" OR "Computerized Tomography" OR "Electron Beam Tomography" OR (("Computed" NEAR/3 ″Tomography") OR ("Computer" NEAR/3 ″Tomography") OR ("Computerised" NEAR/3 ″Tomography") OR ("Computerized" NEAR/3 ″Tomography") OR ("Electron" NEAR/3 ″Beam" NEAR/3 ″Tomography") OR ("Computed" NEAR/3 ″Tomographic") OR ("Computer" NEAR/3 ″Tomographic") OR ("Computerised" NEAR/3 ″Tomographic") OR ("Computerized" NEAR/3 ″Tomographic") OR ("Electron" NEAR/3 ″Beam" NEAR/3 ″Tomographic"))) OR MW("Nuclear Magnetic Resonance Imaging" OR "Magnetic Resonance Imaging" OR "MRI" OR "MR Imaging" OR "MR Image" OR "MR Images" OR "Chemical Shift Imaging" OR "Magnetic Resonance Image" OR "Magnetic Resonance Images" OR "MR Tomographic" OR "MR Tomography" OR "NMR Imaging" OR "NMR Tomography" OR "NMR Tomographic" OR "Proton Spin Tomography" OR "Spin Echo Imaging" OR (("Magnetic" NEAR/3 ″Resonance" NEAR/3 ″Imaging") OR ("MR" NEAR/3 ″Imaging") OR ("MR" NEAR/3 ″Image") OR ("MR" NEAR/3 ″Images") OR ("Chemical" NEAR/3 ″Shift" NEAR/3 ″Imaging") OR ("Magnetic" NEAR/3 ″Resonance" NEAR/3 ″Image") OR ("Magnetic" NEAR/3 ″Resonance" NEAR/3 ″Images") OR ("MR" NEAR/3 ″Tomographic") OR ("MR" NEAR/3 ″Tomography") OR ("NMR" NEAR/3 ″Imaging") OR ("NMR" NEAR/3 ″Tomography") OR ("NMR" NEAR/3 ″Tomographic") OR ("Proton" NEAR/3 ″Spin" NEAR/3 ″Tomography") OR ("Proton" NEAR/3 ″Spin" NEAR/3 ″Tomographic") OR ("Spin" NEAR/3 ″Echo" NEAR/3 ″Imaging")) OR "computer assisted tomography" OR "CT" OR "CAT Scan" OR "CAT Scans" OR "Computed Tomography" OR "Computer Tomography" OR "Computerised Tomography" OR "Computerized Tomography" OR "Electron Beam Tomography" OR (("Computed" NEAR/3 ″Tomography") OR ("Computer" NEAR/3 ″Tomography") OR ("Computerised" NEAR/3 "Tomography") OR ("Computerized" NEAR/3 "Tomography") OR ("Electron" NEAR/3 "Beam" NEAR/3 "Tomography") OR ("Computed" NEAR/3 "Tomographic") OR ("Computer" NEAR/3 "Tomographic") OR ("Computerised" NEAR/3 "Tomographic") OR ("Computerized" NEAR/3 "Tomographic") OR ("Electron" NEAR/3 "Beam" NEAR/3 "Tomographic"))) OR AB("Nuclear Magnetic Resonance Imaging" OR "Magnetic Resonance Imaging" OR "MRI" OR "MR Imaging" OR "MR Image" OR "MR Images" OR "Chemical Shift Imaging" OR "Magnetic Resonance Image" OR "Magnetic Resonance Images" OR "MR Tomographic" OR "MR Tomography" OR "NMR Imaging" OR "NMR Tomography" OR "NMR Tomographic" OR "Proton Spin Tomography" OR "Spin Echo Imaging" OR (("Magnetic" NEAR/3 "Resonance" NEAR/3 "Imaging") OR ("MR" NEAR/3 "Imaging") OR ("MR" NEAR/3 "Image") OR ("MR" NEAR/3 "Images") OR ("Chemical" NEAR/3 "Shift" NEAR/3 "Imaging") OR ("Magnetic" NEAR/3 "Resonance" NEAR/3 "Image") OR ("Magnetic" NEAR/3 "Resonance" NEAR/3 "Images") OR ("MR" NEAR/3 "Tomographic") OR ("MR" NEAR/3 "Tomography") OR ("NMR" NEAR/3 "Imaging") OR ("NMR" NEAR/3 "Tomography") OR ("NMR" NEAR/3 "Tomographic") OR ("Proton" NEAR/3 "Spin" NEAR/3 "Tomography") OR ("Proton" NEAR/3 "Spin" NEAR/3 "Tomographic") OR ("Spin" NEAR/3 "Echo" NEAR/3 "Imaging")) OR "computer assisted tomography" OR "CT" OR "CAT Scan" OR "CAT Scans" OR "Computed Tomography" OR "Computer Tomography" OR "Computerised Tomography" OR "Computerized Tomography" OR "Electron Beam Tomography" OR (("Computed" NEAR/3 "Tomography") OR ("Computer" NEAR/3 "Tomography") OR ("Computerised" NEAR/3 "Tomography") OR ("Computerized" NEAR/3 "Tomography") OR ("Electron" NEAR/3 "Beam" NEAR/3 "Tomography") OR ("Computed" NEAR/3 "Tomographic") OR ("Computer" NEAR/3 "Tomographic") OR ("Computerised" NEAR/3 "Tomographic") OR ("Computerized" NEAR/3 "Tomographic") OR ("Electron" NEAR/3 "Beam" NEAR/3 "Tomographic")))) AND TI("dual energy X ray absorptiometry" OR "dexa" OR "DXA" OR "dual energy Xray absorptiometry" OR "dual energy Xray absorp*" OR "dual energy X ray absorptiometry" OR "dual energy X ray absorp*" OR "dual energy Xray" OR "dual energy Xray*" OR "dual energy X ray" OR "dual energy X rays" OR (("dual" NEAR/3 ″energy" NEAR/3 ″Xray" NEAR/3 ″absorptiometry") OR ("dual" NEAR/3 ″energy" NEAR/3 ″X" NEAR/3 ″ray" NEAR/3 ″absorptiometry") OR ("dual" NEAR/3 ″energy" NEAR/3 ″Xray") OR ("dual" NEAR/3 ″energy" NEAR/3 ″X" NEAR/3 ″ray") OR ("dual" NEAR/3 ″energy" NEAR/3 ″X" NEAR/3 ″rays"))) AND (TI("Lumbar Vertebra" OR "Lumbar Spine" OR "lumbar" OR "low back" OR "lower back" OR "Hip" OR "Hip" OR "Hips" OR "Femur" OR "Femur" OR "vertebral") OR MW("Lumbar Vertebra" OR "Lumbar Spine" OR "lumbar" OR "low back" OR "lower back" OR "Hip" OR "Hip" OR "Hips" OR "Femur" OR "Femur" OR "vertebral") OR AB("Lumbar Vertebra" OR "Lumbar Spine" OR "lumbar" OR "low back" OR "lower back" OR "Hip" OR "Hip" OR "Hips" OR "Femur" OR "Femur" OR "vertebral"))) OR (TI("bone quality" OR "bone quality" OR "Bone Density" OR "bone density" OR "Bone Strength" OR "Bone structure" OR "Bone structure" OR (("bone" NEAR/3 ″quality") OR ("bone" NEAR/3 ″density") OR ("bones" NEAR/3 ″quality") OR ("bones" NEAR/3 ″density") OR ("Bone" NEAR/3 ″structure") OR ("Bone" NEAR/3 ″structures"))) AND (TI("Nuclear Magnetic Resonance Imaging" OR "Magnetic Resonance Imaging" OR "MRI" OR "MR Imaging" OR "MR Image" OR "MR Images" OR "Chemical Shift Imaging" OR "Magnetic Resonance Image" OR "Magnetic Resonance Images" OR "MR Tomographic" OR "MR Tomography" OR "NMR Imaging" OR "NMR Tomography" OR "NMR Tomographic" OR "Proton Spin Tomography" OR "Spin Echo Imaging" OR (("Magnetic" NEAR/3 ″Resonance" NEAR/3 ″Imaging") OR ("MR" NEAR/3 ″Imaging") OR ("MR" NEAR/3 ″Image") OR ("MR" NEAR/3 ″Images") OR ("Chemical" NEAR/3 ″Shift" NEAR/3 ″Imaging") OR ("Magnetic" NEAR/3 ″Resonance" NEAR/3 ″Image") OR ("Magnetic" NEAR/3 ″Resonance" NEAR/3 ″Images") OR ("MR" NEAR/3 ″Tomographic") OR ("MR" NEAR/3 ″Tomography") OR ("NMR" NEAR/3 ″Imaging") OR ("NMR" NEAR/3 ″Tomography") OR ("NMR" NEAR/3 ″Tomographic") OR ("Proton" NEAR/3 ″Spin" NEAR/3 ″Tomography") OR ("Proton" NEAR/3 ″Spin" NEAR/3 ″Tomographic") OR ("Spin" NEAR/3 ″Echo" NEAR/3 ″Imaging")) OR "computer assisted tomography" OR "CT" OR "CAT Scan" OR "CAT Scans" OR "Computed Tomography" OR "Computer Tomography" OR "Computerised Tomography" OR "Computerized Tomography" OR "Electron Beam Tomography" OR (("Computed" NEAR/3 ″Tomography") OR ("Computer" NEAR/3 ″Tomography") OR ("Computerised" NEAR/3 ″Tomography") OR ("Computerized" NEAR/3 ″Tomography") OR ("Electron" NEAR/3 ″Beam" NEAR/3 ″Tomography") OR ("Computed" NEAR/3 ″Tomographic") OR ("Computer" NEAR/3 ″Tomographic") OR ("Computerised" NEAR/3 ″Tomographic") OR ("Computerized" NEAR/3 ″Tomographic") OR ("Electron" NEAR/3 ″Beam" NEAR/3 ″Tomographic"))) OR MW("Nuclear Magnetic Resonance Imaging" OR "Magnetic Resonance Imaging" OR "MRI" OR "MR Imaging" OR "MR Image" OR "MR Images" OR "Chemical Shift Imaging" OR "Magnetic Resonance Image" OR "Magnetic Resonance Images" OR "MR Tomographic" OR "MR Tomography" OR "NMR Imaging" OR "NMR Tomography" OR "NMR Tomographic" OR "Proton Spin Tomography" OR "Spin Echo Imaging" OR (("Magnetic" NEAR/3 ″Resonance" NEAR/3 ″Imaging") OR ("MR" NEAR/3 ″Imaging") OR ("MR" NEAR/3 ″Image") OR ("MR" NEAR/3 ″Images") OR ("Chemical" NEAR/3 ″Shift" NEAR/3 ″Imaging") OR ("Magnetic" NEAR/3 ″Resonance" NEAR/3 ″Image") OR ("Magnetic" NEAR/3 ″Resonance" NEAR/3 ″Images") OR ("MR" NEAR/3 ″Tomographic") OR ("MR" NEAR/3 ″Tomography") OR ("NMR" NEAR/3 ″Imaging") OR ("NMR" NEAR/3 ″Tomography") OR ("NMR" NEAR/3 ″Tomographic") OR ("Proton" NEAR/3 ″Spin" NEAR/3 ″Tomography") OR ("Proton" NEAR/3 ″Spin" NEAR/3 ″Tomographic") OR ("Spin" NEAR/3 ″Echo" NEAR/3 ″Imaging")) OR "computer assisted tomography" OR "CT" OR "CAT Scan" OR "CAT Scans" OR "Computed Tomography" OR "Computer Tomography" OR "Computerised Tomography" OR "Computerized Tomography" OR "Electron Beam Tomography" OR (("Computed" NEAR/3 ″Tomography") OR ("Computer" NEAR/3 ″Tomography") OR ("Computerised" NEAR/3 ″Tomography") OR ("Computerized" NEAR/3 ″Tomography") OR ("Electron" NEAR/3 ″Beam" NEAR/3 ″Tomography") OR ("Computed" NEAR/3 ″Tomographic") OR ("Computer" NEAR/3 ″Tomographic") OR ("Computerised" NEAR/3 ″Tomographic") OR ("Computerized" NEAR/3 ″Tomographic") OR ("Electron" NEAR/3 ″Beam" NEAR/3 ″Tomographic"))) OR AB("Nuclear Magnetic Resonance Imaging" OR "Magnetic Resonance Imaging" OR "MRI" OR "MR Imaging" OR "MR Image" OR "MR Images" OR "Chemical Shift Imaging" OR "Magnetic Resonance Image" OR "Magnetic Resonance Images" OR "MR Tomographic" OR "MR Tomography" OR "NMR Imaging" OR "NMR Tomography" OR "NMR Tomographic" OR "Proton Spin Tomography" OR "Spin Echo Imaging" OR (("Magnetic" NEAR/3 ″Resonance" NEAR/3 ″Imaging") OR ("MR" NEAR/3 ″Imaging") OR ("MR" NEAR/3 ″Image") OR ("MR" NEAR/3 ″Images") OR ("Chemical" NEAR/3 ″Shift" NEAR/3 ″Imaging") OR ("Magnetic" NEAR/3 ″Resonance" NEAR/3 ″Image") OR ("Magnetic" NEAR/3 ″Resonance" NEAR/3 ″Images") OR ("MR" NEAR/3 ″Tomographic") OR ("MR" NEAR/3 ″Tomography") OR ("NMR" NEAR/3 ″Imaging") OR ("NMR" NEAR/3 ″Tomography") OR ("NMR" NEAR/3 ″Tomographic") OR ("Proton" NEAR/3 ″Spin" NEAR/3 ″Tomography") OR ("Proton" NEAR/3 ″Spin" NEAR/3 ″Tomographic") OR ("Spin" NEAR/3 ″Echo" NEAR/3 ″Imaging")) OR "computer assisted tomography" OR "CT" OR "CAT Scan" OR "CAT Scans" OR "Computed Tomography" OR "Computer Tomography" OR "Computerised Tomography" OR "Computerized Tomography" OR "Electron Beam Tomography" OR (("Computed" NEAR/3 ″Tomography") OR ("Computer" NEAR/3 ″Tomography") OR ("Computerised" NEAR/3 ″Tomography") OR ("Computerized" NEAR/3 ″Tomography") OR ("Electron" NEAR/3 ″Beam" NEAR/3 ″Tomography") OR ("Computed" NEAR/3 ″Tomographic") OR ("Computer" NEAR/3 ″Tomographic") OR ("Computerised" NEAR/3 ″Tomographic") OR ("Computerized" NEAR/3 ″Tomographic") OR ("Electron" NEAR/3 ″Beam" NEAR/3 ″Tomographic")))) AND (TI("dual energy X ray absorptiometry" OR "dexa" OR "DXA" OR "dual energy Xray absorptiometry" OR "dual energy Xray absorp*" OR "dual energy X ray absorptiometry" OR "dual energy X ray absorp*" OR "dual energy Xray" OR "dual energy Xray*" OR "dual energy X ray" OR "dual energy X rays" OR (("dual" NEAR/3 ″energy" NEAR/3 ″Xray" NEAR/3 ″absorptiometry") OR ("dual" NEAR/3 ″energy" NEAR/3 ″X" NEAR/3 ″ray" NEAR/3 ″absorptiometry") OR ("dual" NEAR/3 ″energy" NEAR/3 ″Xray") OR ("dual" NEAR/3 ″energy" NEAR/3 ″X" NEAR/3 ″ray") OR ("dual" NEAR/3 ″energy" NEAR/3 ″X" NEAR/3 ″rays"))) OR MW("dual energy X ray absorptiometry" OR "dexa" OR "DXA" OR "dual energy Xray absorptiometry" OR "dual energy Xray absorp*" OR "dual energy X ray absorptiometry" OR "dual energy X ray absorp*" OR "dual energy Xray" OR "dual energy Xray*" OR "dual energy X ray" OR "dual energy X rays" OR (("dual" NEAR/3 ″energy" NEAR/3 ″Xray" NEAR/3 ″absorptiometry") OR ("dual" NEAR/3 ″energy" NEAR/3 ″X" NEAR/3 ″ray" NEAR/3 ″absorptiometry") OR ("dual" NEAR/3 ″energy" NEAR/3 ″Xray") OR ("dual" NEAR/3 ″energy" NEAR/3 ″X" NEAR/3 ″ray") OR ("dual" NEAR/3 ″energy" NEAR/3 ″X" NEAR/3 ″rays"))) OR AB("dual energy X ray absorptiometry" OR "dexa" OR "DXA" OR "dual energy Xray absorptiometry" OR "dual energy Xray absorp*" OR "dual energy X ray absorptiometry" OR "dual energy X ray absorp*" OR "dual energy Xray" OR "dual energy Xray*" OR "dual energy X ray" OR "dual energy X rays" OR (("dual" NEAR/3 ″energy" NEAR/3 ″Xray" NEAR/3 ″absorptiometry") OR ("dual" NEAR/3 ″energy" NEAR/3 ″X" NEAR/3 ″ray" NEAR/3 ″absorptiometry") OR ("dual" NEAR/3 ″energy" NEAR/3 ″Xray") OR ("dual" NEAR/3 ″energy" NEAR/3 ″X" NEAR/3 ″ray") OR ("dual" NEAR/3 ″energy" NEAR/3 ″X" NEAR/3 ″rays")))) AND TI("Lumbar Vertebra" OR "Lumbar Spine" OR "lumbar" OR "low back" OR "lower back" OR "Hip" OR "Hip" OR "Hips" OR "Femur" OR "Femur" OR "vertebral"))) **NOT TI("veterinary" OR "rabbit" OR "rabbits" OR "animal" OR "animals" OR "mouse" OR "mice" OR "rodent" OR "rodents" OR "rat" OR "rats" OR "pig" OR "pigs" OR "porcine" OR "horse" OR "horses" OR "equine" OR "cow" OR "cows" OR "bovine" OR "goat" OR "goats" OR "sheep" OR "ovine" OR "canine" OR "dog" OR "dogs" OR "feline" OR "cat" OR "cats")** NOT (TI("Case Report") OR MW("case report")))
